# Assessment of the impact of phenylketonuria and its treatment on quality of life of patients and parents from seven European countries

**DOI:** 10.1186/s13023-015-0294-x

**Published:** 2015-06-18

**Authors:** Annet M Bosch, Alberto Burlina, Amy Cunningham, Esther Bettiol, Flavie Moreau-Stucker, Ekaterina Koledova, Khadra Benmedjahed, Antoine Regnault

**Affiliations:** Department of Paediatrics, Division of Metabolic Disorders, Academic Medical Centre, University Hospital of Amsterdam, Amsterdam, The Netherlands; Division of Metabolic Disorders, Department of Paediatrics, University Hospital of Padova, Padova, Italy; Hayward Genetics Center, Tulane University School of Medicine, New Orleans, LA USA; Infection Control Program, University of Geneva Hospitals and Faculty of Medicine, Geneva, Switzerland; EMD Serono Inc, Billerica, MA USA; Mapi, Health Economics & Outcomes Research and Strategic Market Access, Lyon, France; Merck KGaA, Darmstadt, Germany

**Keywords:** Rare disease, Phenylketonuria, Questionnaires, Health-related quality of life

## Abstract

**Background:**

The strict and demanding dietary treatment and mild cognitive abnormalities seen in PKU treated from a young age can be expected to affect the health-related quality of life (HRQoL) of patients and their families. Our aim was to describe the HRQoL of patients with PKU from a large international study, using generic HRQoL measures and an innovative PKU-specific HRQoL questionnaire (PKU-QOL). Analyses were exploratory, performed post-hoc on data collected primarily to validate the PKU-QOL.

**Methods:**

A multicentre, prospective, non-interventional, observational study conducted in France, Germany, Italy, The Netherlands, Spain, Turkey and the UK. Patients diagnosed with PKU aged ≥9 years old and treated with a Phe-restricted diet and/or Phe-free amino acid protein supplements and/or pharmacological therapy were included in the study; parents of at least one patient with PKU aged <18 years were also included. HRQoL was assessed by generic measures (Pediatric Quality-of-Life Inventory; Medical Outcome Survey 36 item Short Form; Child Health Questionnaire 28 item Parent Form) and the newly developed PKU-QOL. Mean generic domain scores were interpreted using published reference values from the general population. PKU-QOL domain scores were described overall and in different subgroups of patients defined according to severity of PKU, overall assessment of patient’s health status by the investigator and treatment with tetrahydrobiopterin (BH_4_).

**Results:**

Data from 559 subjects were analysed: 306 patients (92 children, 110 adolescents, 104 adults) and 253 parents. Mean domain scores of generic measures in the study were comparable to the general population. The highest PKU-QOL impact scores (indicating greater impact) were for emotional impact of PKU, anxiety about blood Phe levels, guilt regarding poor adherence to dietary restrictions or Phe-free amino acid supplement intake and anxiety regarding blood Phe levels during pregnancy. Patients with mild/moderate PKU and those receiving BH_4_ reported lower practical and emotional impacts of the diet and Phe-free amino acid supplement intake.

**Conclusion:**

Patients with PKU showed good HRQoL in the study, both with the generic and PKU-specific measures. Negative impacts of PKU on a patient’s life, including the emotional impact of PKU and its management, was delineated by the PKU-QOLs across all age groups.

**Electronic supplementary material:**

The online version of this article (doi:10.1186/s13023-015-0294-x) contains supplementary material, which is available to authorized users.

## Background

Phenylketonuria (PKU; MIM 261600) is a rare inherited disorder of phenylalanine (Phe) metabolism caused by a deficiency of the enzyme phenylalanine hydroxylase (PAH; EC 1.14.16.1). In the absence or deficiency of PAH, the essential amino acid phenylalanine cannot be sufficiently hydroxylated to tyrosine (Tyr). This results in increased blood concentrations of Phe and toxic accumulation in the brain. Untreated PKU results in severe intellectual disability and neurological abnormalities [1]. The incidence in Europe is estimated to be 1:10.000 [2].

The implementation of neonatal screening programs in the 1960s enabled early diagnosis and initiation of treatment for PKU, thus successfully preventing mental disability. Treatment for PKU includes a life-long diet restricted in Phe that is supported nutritionally with a Phe-free amino acid supplement (i.e., medical food, metabolic formula, amino acid mixtures). Maintaining low blood Phe levels by strict adherence to the diet is the strongest determinant for a good cognitive outcome [1, 3, 4].

However, the management of PKU is complex and can be burdensome. A Phe-restricted diet excludes many foods naturally high in protein (such as dairy products, meat, fish, eggs and bread) and the Phe-free amino acid supplements required for nutritional completeness of the diet are considered unpalatable by many patients. Additionally, specialty low-protein foods necessary for adequate quantity and variety of food within the very restricted diet may be poorly accepted and create a financial burden. Adherence to diet therapy is essential, especially during early childhood years, but is often problematic and an association of the diet with adverse feeding behaviours in young patients has been reported [5–8]. Recent and ongoing development of pharmacological treatments may allow modification of dietary restriction in some patients, but do not yet consistently allow discontinuation of traditional diet therapy.

While patients treated continuously from an early age generally have a favourable outcome with normal overall development, subtle abnormalities have been demonstrated [9].

The IQ of patients treated from an early age has been demonstrated to be within the normal range but below the population mean [10], and executive function deficits, associated with concurrent high blood Phe levels, are observed in both children (who suffer the strongest effect as demonstrated in a meta-analysis [11] and adults [12–16]. Furthermore, high blood Phe levels were demonstrated to have a direct negative effect on mood in a crossover, double-blind placebo-controlled study that used the Profile of Mood States (POMS) questionnaire [16].

Both the strict and demanding therapy and the mild cognitive abnormalities seen in PKU treated from an early age can be expected to affect the quality of life of patients and their families. However, studies evaluating the Health Related Quality of Life (HRQoL) of patients with PKU have demonstrated an HRQoL comparable to that of the general population [17–20], with the exception of a lower HRQoL demonstrated in a group of Italian children [21] and a lower score on the cognitive scale in Dutch adults [22]. These unexpected normal scores in most studies may result from the insufficient sensitivity of the presently available generic HRQoL measurement tools in detecting the specific problems of patients with PKU. This is confirmed by the fact that only a study using a partially validated PKU-specific questionnaire demonstrated an improvement in patients’ HRQoL after the start of BH_4_, which allowed relaxation of the diet in some of the patients [22, 23]. Of note, in these studies, HRQoL was assessed for about 50 patients (maximum being 69 in Demirdas et al.), always from one single country.

In order to evaluate the effectiveness of therapeutic and supportive interventions for patients with PKU, a PKU-specific questionnaire is warranted. This paper aims to describe the HRQoL of a large and international panel of patients with PKU and their parents via the administration of an innovative PKU specific questionnaire. The conceptual model, plus details of the scaling/scoring structure and validation of this new questionnaire, are reported elsewhere [24].

## Methods

### Study design

A multicentre, prospective, non-interventional, observational study was conducted in 34 sites in France, Germany, Italy, The Netherlands, Spain, Turkey and the UK from December 2011 to November 2012. The primary objective of the study was to finalize and validate the newly developed PKU-specific Quality-of-Life questionnaire (PKU-QOL). The results related to this primary objective are reported elsewhere [24].

A secondary objective of the study, which is reported here, was to describe HRQoL as measured for the first time by instruments designed to be sensitive to the particular and sometimes subtle issues characteristic of the PKU population. As this was the secondary objective, this paper presents exploratory analyses performed post-hoc on data collected primarily for the purpose of validating the PKU-QOL.

Patients included in the study were aged ≥9 years old, diagnosed with PKU and treated for PKU with a Phe-restricted diet and/or Phe-free amino acid supplements (medical food, metabolic formula) and/or pharmacological therapy. Patients in this study were categorized into three groups: Children (9–11 years), Adolescents (12–17 years) and Adults (≥18 years). Parents of at least one patient aged <18 years old treated for PKU with a Phe-restricted diet and/or Phe-free amino acid supplements and/or pharmacological therapy were also included.

The study was performed in accordance with good clinical practice and in compliance with local regulatory requirements. The appropriate national authorities and institutional review boards approved the protocol before study commencement. All patients or their legally authorized representatives provided written informed consent.

The validation study protocol was submitted to the Conseil National de l’ordre de Médecins (CNOM) in France, to the Ethik-Kommission an der Medizinischen Fakultät der Universität Leipzig, Ethikkommission der Medizinischen Fakultät Heidelberg, Ethik-Kommission der MHH, Ethik-Kommission des Fachbereichs Medizin der Johann Wolfgang Goethe- Universität, Ethik-Kommission der Ärztekammer Westfalen-Lippe und der medizinischen Fakultät der Westfälischen Wilhelms Universität Münster and Ethik-Kommission bei der Landesärztekammer Baden-Württemberg in Germany, to the Comitato Etico della Azienda Ospedaliero-Universitaria di Bologna, Comitato Etico per la Sperimentazione Clincal della Provincia di Vicenza, Comitato Etico per la Sperimentazione della Azienda Ospedaliera di Padova, Comitato Etico della ASL NA/1 di Napoli, Comitato di Etica dell ‘IRCCS Istituto Giannina Gaslini de Genova’ and Comitato Etico Dell ‘Azienda Policlinico Umberto I Di Roma’ in Italy, to the Academisch Medisch Centrum Amsterdam, Universitair Medisch Centrum Groningen and Academisch Ziekenhuis Maastricht (AZM) in the Netherlands, to CCAA Andalucía, CCAA Galicia, CCAA Aragón, CCAA Baleares, CCAA Pais Vasco and H. Univ Virgen del Rocio in Spain, to Istanbul University Cerrahpasa Medical Faculty Ethic Committee in Turkey, to R&D of Glasgow Royal Infirmary, R&D of Central Manchester University Hospitals NHS Foundation Trust, R&D of University Hospitals Bristol and Guy’s & St Thomas’ Hospital NHS Foundation Trust in the UK.

### Health-Related Quality of life measures

HRQoL was assessed in the study by both generic and PKU-specific HRQoL measures. The measures were adapted to the respondent: children and adolescents completed the Pediatric Quality-of-Life Inventory (PedsQL) and the Child or Adolescent version of the PKU-QOL; adult patients completed the Medical Outcome Survey 36 item Short Form (SF-36) and the Adult version of the PKU-QOL; and parents of patients with PKU completed the Child Health Questionnaire 28 item Parent Form (CHQ-PF28) and the Parent version of the PKU-QOL.

The PKU-QOL is a questionnaire designed to specifically assess the impact of PKU on all aspects of PKU patients’ life, including: PKU symptoms; the practical, social and emotional impact of PKU on patient’s life; the impact of low-protein dietary restrictions; and the impact of Phe-free amino acid supplement intake (additional detail for the questionnaires can be found at http://www.proqolid.org). It has been developed and validated according to standard methods involving patients’ and clinicians’ input at all stages [24]. It exists in four versions: three to be completed by patients with PKU and adapted to their age (Children, Adolescents and Adults); and one to be completed by parents of patients with PKU. Depending on the version, the number of items included in the PKU-QOL ranges between 40 (Child version) and 65 (Adult version), which allow the assessment of between 28 and 35 domain scores. The domain scores for each version can be found in Table 1. PKU-QOL domain scores were interpreted based on the domain content as follows: scores of <26 indicate little or no impact (or very limited or no symptoms for symptom scores), scores of >25 and <51 indicate a moderate impact (or moderate symptoms), scores of >50 and <76 indicate major impact (or severe and/or frequent symptom), scores of >75 indicate severe impact (or very severe and/or very frequent symptoms). Self-rated health status (a subjective comparison of health with others of the same age, made by the respondent) was rated as poor (PKU-QOL score = 4), fair (PKU-QOL score = 3), good (PKU-QOL score = 2), very good (PKU-QOL score = 1) or excellent (PKU-QOL score = 0) as part of the Adolescent, Adult and Parent PKU-QOLs.

**Table 1:** PKU-QOL scores in the study samples

PKU-QOL domains – Median (Q1-Q3)	Child Sample	Adolescent sample	Adult sample	Parent sample
	(*N* = 92)	(*N* = 110)	(*N* = 104)	(*N* = 253)
Symptoms				
Self-rated health status	-	50.0 (25.0–50.0)	25.0 (25.0–50.0)	-
Child health status	-	-	-	25.0 (0.0–50.0)
Headaches	0.0 (0.0–25.0)	0.0 (0.0–25.0)	25.0 (0.0–50.0)	0.0 (0.0–25.0)
Stomach aches	0.0 (0.0–25.0)	0.0 (0.0–25.0)	0.0 (0.0–25.0)	0.0 (0.0–25.0)
Tiredness	25.0 (25.0–50.0)	50.0 (25.0–50.0)	50.0 (25.0–50.0)	25.0 (0.0–50.0)
Trembling hands	-	-	0.0 (0.0–25.0)	-
Irritability	0.0 (0.0–25.0)	25.0 (0.0–50.0)	25.0 (0.0–50.0)	25.0 (25.0–50.0)
Aggressiveness	0.0 (0.0–25.0)	0.0 (0.0–25.0)	0.0 (0.0–25.0)	0.0 (0.0–25.0)
Moodiness	0.0 (0.0–25.0)	25.0 (0.0–50.0)	25.0 (0.0–50.0)	25.0 (0.0–50.0)
Sadness	0.0 (0.0–25.0)	0.0 (0.0–25.0)	25.0 (0.0–50.0)	25.0 (0.0–25.0)
Anxiety	0.0 (0.0–0.0)	0.0 (0.0–25.0)	0.0 (0.0–50.0)	0.0 (0.0–25.0)
Lack of concentration	0.0 (0.0–25.0))	0.0 (0.0–25.0)	25.0 (0.0–50.0)	25.0 (0.0–50.0)
Slow thinking	25.0 (0.0–50.0)	0.0 (0.0–25.0)	0.0 (0.0–25.0)	0.0 (0.0–50.0)
PKU in general				
Emotional impact of PKU	33.3 (16.7–50.0)	30.0 (20.0–40.0)	45.0 (25.0–60.0)	37.5 (25.0–62.5)
Practical impact of PKU	0.0 (0.0–12.5)	8.3 (0.0–25.0)	12.5 (6.3–25.0)	12.5 (0.0–25.0)
Social impact of PKU	16.7 (8.3–25.0)	16.7 (8.3–25.0)	16.7 (6.3–25.0)	12.5 (5.0–25.0)
Overall impact of PKU	18.8 (12.5–31.3)	20.0 (12.5–30.0)	27.1 (18.8–39.6)	21.4 (11.5–35.0)
Anxiety – blood test	6.3 (0.0–12.5)	0.0 (0.0–12.5)	0.0 (0.0–12.5)	12.5 (0.0–37.5)
Impact of child anxiety – blood test	-	-	-	12.5 (0.0–37.5)
Anxiety – blood Phe levels	50.0 (25.0–75.0)	25.0 (0.0–50.0)	25.0 (25.0–50.0)	50.0 (25.0–75.0)
Anxiety – blood Phe levels during pregnancy	-	-	100.0 (75.0–100.0)^a^	-
Financial impact of PKU	-	-	0.0 (0.0–25.0)	25.0 (0.0–50.0)
Information on PKU	-	-	25.0 (25.0–50.0)	25.0 (0.0–50.0)
Phe-free amino acid supplement administration				
Adherence to supplements	0.0 (0.0–12.5)	6.3 (0.0–25.0)	16.7 (0.0–33.3)	0.0 (0.0–25.0)
Management of supplements	-	-	-	0.0 (0.0–25.0)
Practical impact of supplements	0.0 (0.0–25.0)	12.5 (0.0–25.0)	18.8 (6.3–37.5)	8.3 (0.0–33.3)
Guilt if poor adherence to supplements	37.5 (0.0–75.0)	25.0 (25.0–75.0)	50.0 (25.0–75.0)	50.0 (25.0–75.0)
Relationships within family because of supplements	0.0 (0.0–0.0)	0.0 (0.0–25.0)	0.0 (0.0–25.0)	0.0 (0.0–25.0)
Taste – supplements	50.0 (0.0–50.0)	50.0 (25.0–50.0)	50.0 (25.0–50.0)	-
Dietary protein restriction				
Food temptations	25.0 (0.0–50.0)	25.0 (0.0–50.0)	37.5 (12.5–50.0)	-
Adherence to dietary protein restriction	0.0 (0.0–12.5)	8.3 (0.0–18.8)	18.8 (0.0–31.3)	0.0 (0.0–25.0)
Management of dietary protein restriction	-	-	-	20.8 (5.0–37.5)
Social impact of dietary protein restriction	15.0 (5.0–30.0)	5.0 (0.0–20.0)	12.5 (4.2–25.0)	-
Practical impact of dietary protein restriction	-	28.6 (14.3–32.1)	35.0 (20.8–50.0)	28.6 (14.3–46.4)
Overall impact of dietary protein restriction	-	17.8 (9.1–25.0)	25.0 (11.5–37.5)	-
Taste – specialty low protein food	25.0 (0.0–25.0)	25.0 (0.0–37.5)	25.0 (25.0–50.0)	-
Food enjoyment	0.0 (0.0–25.0)	0.0 (0.0–25.0)	25.0 (0.0–50.0)	25.0 (0.0–25.0)
Guilt if dietary protein restriction not followed	50.0 (0.0–100.0)	50.0 (25.0–75.0)	50.0 (25.0–75.0)	50.0 (25.0–75.0)
Overall difficulty following dietary protein restriction	0.0 (0.0–25.0)	0.0 (0.0–25.0)	25.0 (0.0–50.0)	-

Note: lower PKU-QOL scores represent more positive outcome; 0 (no impact/no symptom) and 100 (extremely severe impact/very frequent symptom)

^a^Female patients only, *n* = 66

The PedsQL is designed to assess HRQoL in children [25–29]. It is applicable for healthy school and community populations, as well as paediatric populations with acute and chronic health conditions. The PedsQL Generic Core Scales include 23 items grouped into four domains: Physical Functioning, Emotional Functioning, Social Functioning, and School Functioning.

The SF-36 is a widely used questionnaire, designed to measure generic health concepts relevant across adult age, disease and treatment groups [30–32]. It is a well-validated measure of general health status and QoL. It includes eight domains: Physical Functioning, Role-Physical, Bodily Pain, General Health, Vitality, Social Functioning, Role-Emotional, and Mental Health. Two summary scores can be calculated based on the eight domain scores: the Standardized Physical Component Scale and the Standardized Mental Component Scale.

The CHQ is a family of generic QoL questionnaires designed for children and their parents [33, 34]. The 28-item parent form of the CHQ (CHQ-PF28) contains the following domains: Emotional and Time Impact on the Parent, Limitations in Family Activities and Family Cohesion.

### Statistical analyses

HRQoL domain scores were computed for both generic and PKU-specific HRQoL measures in each group (Children, Adolescent, Adults, Parents). Mean generic domain scores obtained in this study were interpreted in light of reference values from published general population samples for the PedsQL [35], the SF-36 [36] and the CHQ-PF28 [37]. Reference values from the US population were used despite the European nature of our sample because they were the only ones consistently available from large samples for all questionnaires. Median and interquartile range for PKU-QOL scores are reported throughout the manuscript to take into account some skewed distributions. Means were also computed for consistency with the scores from the generic instruments, and are reported in the supplementary materials.

PKU-QOL domain scores were then compared between different subgroups of patients defined according to severity of PKU (defined as mild/moderate [Phe level at diagnosis 600–1200 μmol/L] vs. classical [Phe level at diagnosis >1200 μmol/L]); overall assessment of patient’s health status as rated by the investigator (i.e., response to the question: “In general, how would you rate the overall health status of your patient?” Poor/Fair/Good/Very good/Excellent), and treatment with tetrahydrobiopterin (BH_4_).

These analyses were secondary analyses of data collected using the PKU-QOL under validation so the sample size of the study was not specifically determined for the results presented here. Also, given the descriptive and explanatory nature of these analyses, no adjustment for multiple testing has been applied, and the p-values provided should be considered in a purely explorative way.

## Results

### Study samples

Of the 617 subjects recruited in the study, 559 returned a PKU-QOL and were used in the analyses: 306 patients with PKU and 253 parents of patients with PKU. The patient sample included 92 children aged 9–11 years, 110 adolescents aged 12–17 years and 104 adults older than 18 years of age. The parent sample included the parents of 201 subjects from the child or adolescent sample, and 52 additional parents of children younger than eight years old.

Detailed demographic and clinical characteristics of the samples are provided in Table 2. Clinician overall assessment of patient’s health status ranged from good to excellent for almost all subjects across all samples. The distribution between male and female subjects was balanced in all study groups, except in the Adult and Parent samples, where the percentage of female subjects was higher. More than six out of 10 subjects had a classical PKU at the time of diagnosis (Phe level >1200 μmol/L). Almost half of the patients (43.5%) only had a medical follow-up (i.e., a follow-up by a GP, paediatrician, endocrinologist, and/or specialized nurse) and 31.7% were followed by a dietician/nutritionist in addition to the medical team. In subjects following a pharmacological treatment, BH_4_ appeared to be the only pharmacological treatment reported for a substantial proportion of patients (14–29%, depending on the sample).

**Table 2:** Characteristics of the samples used in the analyses

		Child sample (*N* = 92)	Adolescent sample (*N* = 110)	Adult sample (*N* = 104)	Parent sample^a^ (*N* = 253)	Young Children sample^b^ (*N* = 52)
Age (years)	n (missing)	90 (2)	110 (0)	101 (3)	244 (9)	52 (0)
	Mean (SD)	9.8 (0.8)	14.5 (1.6)	25.8 (6.6)	41.6 (6.5)	4.4 (2.5)
	Min – Max	9.0–11.0	12.0–17.0	18.0–45.0	24.0–66.0	0.0–8.0
Gender	Male	43 (46.7)	56 (50.9)	38 (36.5)	69 (27.3)	28 (53.8)
n (%)						
	Female	47 (51.1)	54 (49.1)	66 (63.5)	183 (72.3)	24 (46.2)
	Missing	2 (2.2)	0 (0.0)	0 (0.0)	1 (0.4)	0 (0.0)
Treated with BH_4_	n (%)	27 (29.3)	27 (24.5)	15 (14.4)	–	13 (25.0)
PKU severity	Classical^c^	66 (71.7)	75 (68.2)	67(64.4)	–	32 (61.5)
n (%)						
	Missing	4 (4.3)	2 (1.8)	0 (0.0)	–	0 (0.0)
Management	Medical only	45 (48.9)	49 (44.5)	39 (37.5)	–	21 (40.4)
n (%)						
	Multi-disciplinary team	45 (48.9)	61 (55.5)	63 (60.6)	–	31 (59.6)
	Missing	2 (2.2)	0 (0.0)	0 (0.0)	–	0 (0.0)
Overall health status rating by clinician	Poor	0 (0.0)	0 (0.0)	0 (0.0)	–	0 (0.0)
	Fair	2 (2.1)	0 (0.0)	3 (2.9)	–	0 (0.0)
n (%)						
	Good	19 (20.7)	38 (34.5)	28 (26.9)	–	7 (13.5)
	Very good	41 (44.6)	42 (38.2)	46 (44.2)	–	18 (34.6)
	Excellent	28 (30.4)	30 (27.3)	26 (25.0)	–	27 (51.9)
	Missing	2 (2.2)	0 (0.0)	1 (1.0)	–	0 (0.0)
Country	France	13 (14.1)	10 (9.1)	18 (17.3)	33 (13.0)	10 (19.2)
n (%)						
	Germany	19 (20.7)	20 (18.2)	21 (20.2)	46 (18.2)	8 (15.4)
	Italy	15 (16.3)	26 (23.6)	22 (21.2)	49 (19.4)	8 (15.4)
	Netherlands	7 (7.6)	10 (9.1)	7 (6.7)	23 (9.1)	6 (11.5)
	Spain	20 (21.7)	20 (18.2)	21 (20.2)	48 (19.0)	8 (15.4)
	Turkey	14 (15.2)	20 (18.2)	8 (7.7)	40 (15.8)	6 (11.5)
	UK	4 (4.3)	4 (3.6)	7 (6.7)	14 (5.5)	6 (11.5)
	Missing	0 (0.0)	0 (0.0)	0 (0.0)	0 (0.0)	0 (0.0)

*SD* standard deviation

^a^Data for the Parent sample relate to the parent and not the children; hence characteristics related to disease are not reported

^b^Children with PKU younger than eight years old for whom a parent-completed PKU-QOL was collected

^c^Classical PKU defined as Phe level at diagnosis >1200 μmol/L

### Description of Health-Related Quality of life of PKU patients

#### HRQoL assessed by generic questionnaires

Scores of the generic QOL instruments, PedsQL, SF-36 and CHQ-PF28 administered to children and adolescents, adults and parents, respectively, are presented in Table 3. All PedsQL scores of children and adolescents with PKU were in the same range (±3points) as those observed in a large health survey conducted in US children aged 8 to 16 years [35], except the Social functioning score which was 7.9 points higher in our sample. In the sample of adults with PKU, SF-36 scores pertaining to the physical domains (Physical functioning, Role-Physical, Bodily Pain, standardized physical component) were higher than those observed in a large survey conducted in the US general population while the observed mean scores pertaining to mental domains (Mental Health, Role-Emotional, standardized Mental Component) were consistently slightly lower. The largest difference for the SF-36 was observed for Physical functioning for which adults with PKU reported a higher score (95.0 ± 10.2) than the general US population (80.6 ± 25.1), suggesting physical functioning is less impacted in these patients than in the general US population. Most CHQ-PF28 scores observed in parents of children and adolescents with PKU were very similar (± 3.5 points) to those reported by a general US population sample. The only CHQ-PF28 domain score with a larger difference was Emotional Parental impact for which parents of children with PKU reported a 10.9 point lower score (70.4 ± 28.8) than the general US population (81.3 ± 18.4), suggesting a greater emotional impact in these parents than in the general US population.

**Table 3:** Scores from generic QOL instruments (PedsQL, SF-36 and CHQ-PF28) in the study samples and in the US general population

Generic QOL instruments	Study samples	US normative data samples
PedsQL scores – Mean (SD)	Child Sample (*N* = 92)	(*N* = 5972)^a^
Physical functioning score	88.9 (10.7)	86.9 (13.9)
Emotional functioning score	78.8 (16.9)	78.2 (18.6)
Social functioning score	91.9 (12.6)	84.0 (17.0)
School functioning score	80.3 (14.0)	80.0 (17.0)
Psychosocial health summary score	83.7 (11.1)	80.7 (14.7)
PedsQL total scale score	85.5 (9.8)	82.9 (13.2)
PedsQL scores – Mean (SD)	Adolescent Sample (*N* = 110)	(*N* = 5972)^a^
Physical functioning score	88.8 (11.1)	86.9 (13.9)
Emotional functioning score	81.0 (16.2)	78.2 (18.6)
Social functioning score	90.9 (14.0)	84.0 (17.0)
School functioning score	77.1 (15.6)	80.0 (17.0)
Psychosocial health summary score	83.1 (11.9)	80.7 (14.7)
PedsQL total scale score	85.1 (10.5)	82.9 (13.2)
SF-36 scores – Mean (SD)	Adult Sample (*N* = 104)	(*N* = 7069)^b^
Physical Functioning	95.0 (10.2)	80.6 (25.1)
Role-Physical	86.2 (19.3)	80.8 (25.5)
General Health	77.8 (18.2)	70.1 (21.3)
Bodily Pain	83.9 (20.5)	70.1 (24.3)
Role-Emotional	83.3 (22.6)	86.3 (22.5)
Mental Health	71.8 (19.1)	75.2 (18.4)
Vitality	61.7 (19.1)	58.8 (20.7)
Social Functioning	84.4 (21.5)	83.7 (23.6)
Mental Component Scale	46.8 (10.8)	49.9 (10.1)
Physical Component Scale	55.9 (5.5)	50.0 (10.0)
CHQ-PF28 – Mean (SD)	Parent Sample (*N* = 253)	(*N* = 391)^c^
Physical Functioning	95.3 (14.3)	95.0 (16.2)
Role/Social Limitations – Physical	96.1 (14.4)	93.7 (19.7)
General Health Perceptions	70.5 (17.6)	74.0 (19.8)
Bodily Pain/Discomfort	83.0 (18.8)	81.3 (19.7)
Parental Impact – Time	87.9 (21.3)	88.4 (20.9)
Parental Impact – Emotional	70.4 (28.8)	81.3 (18.4)
Role/Social Limitations – Emotional/Behavioral	90.9 (21.3)	92.5 (19.1)
Self Esteem	79.4 (19.1)	80.1 (19.1)
Mental Health	77.0 (19.0)	79.7 (15.5)
Behavior	73.1 (18.9)	70.8 (18.7)
Family Activities	88.3 (19.7)	91.1 (18.9)
Family Cohesion	72.5 (22.2)	72.4 (21.6)
Physical Summary Score	53.0 (7.8)	53.2 (9.5)
Psychosocial Summary Score	49.3 (10.6)	51.1 (9.6)

*SD* standard deviation, *PedsQL* pediatric quality of life inventory, *SF-36* 36-item short form, *CHQ-PF28* child health questionnaire parent form 28 items, *US* United States

Note: higher scores represent more positive outcome for each instrument

^a^Data from Varni et al. 2003 [35]

^b^Data from Ware et al. 2007 [36]

^c^Data from HealthActCHQ. 2008 [37]

#### HRQoL assessed by the PKU-QOL

PKU-QOL scores for each sample are presented in Table 1. Overall, adolescents and adults rated their overall health status as good (38.2% and 40.4% of patients, respectively) or very good (23.6% and 30.8% of patients, respectively), compared with others of their age. A similar finding was also made in parents reporting on the health status of their child. For all age groups, the highest observed median symptom score for self-reported patient versions was for tiredness (25.0 [25.0–50.0] in the Child, 50.0 [25.0–50.0] in the Adolescent and 50.0 [25.0–50.0] in the Adult versions – all indicating moderate symptoms). The observed median tiredness score in the Parent version reporting about their child’s HRQoL (25.0 [0.0–50.0]) was just below the score indicating moderate symptoms. For the Parent version, the median symptom scores for child health status (25.0 [0.0–50.0]), irritability (25.0 [25.0–50.0]), moodiness (25.0 [0.0–50.0]), sadness (25.0 [0.0–25.0]) and lack of concentration (25.0 [0.0–50.0]) also fell just short of the score interpreted as indicating a moderate level of symptoms.

The highest PKU-QOL impact scores were the ones measuring the emotional impact of PKU and its management (emotional impact of PKU, anxiety about blood Phe levels, guilt related to poor adherence to dietary restrictions or Phe-free amino acid supplement intake). The highest median score (i.e., the highest impact of PKU), across all scores from all versions, was for the score measuring anxiety regarding blood Phe levels during pregnancy in the Adult version of the PKU questionnaire with a median score of 100.0 (75.0–100.0).

In all patient versions, the score assessing the impact of the taste of Phe-free amino acid supplements was also one of the highest, indicating the poor palatability and taste acceptance for these products (50.0 [0.0–50.0] in the Child, 50.0 [25.0–50.0] in the Adolescent and 50.0 [25.0–50.0] in the Adult versions).

### PKU-QOL scores between subgroups of patients

#### PKU-QOL scores according to severity of PKU

**Fig. 1:**
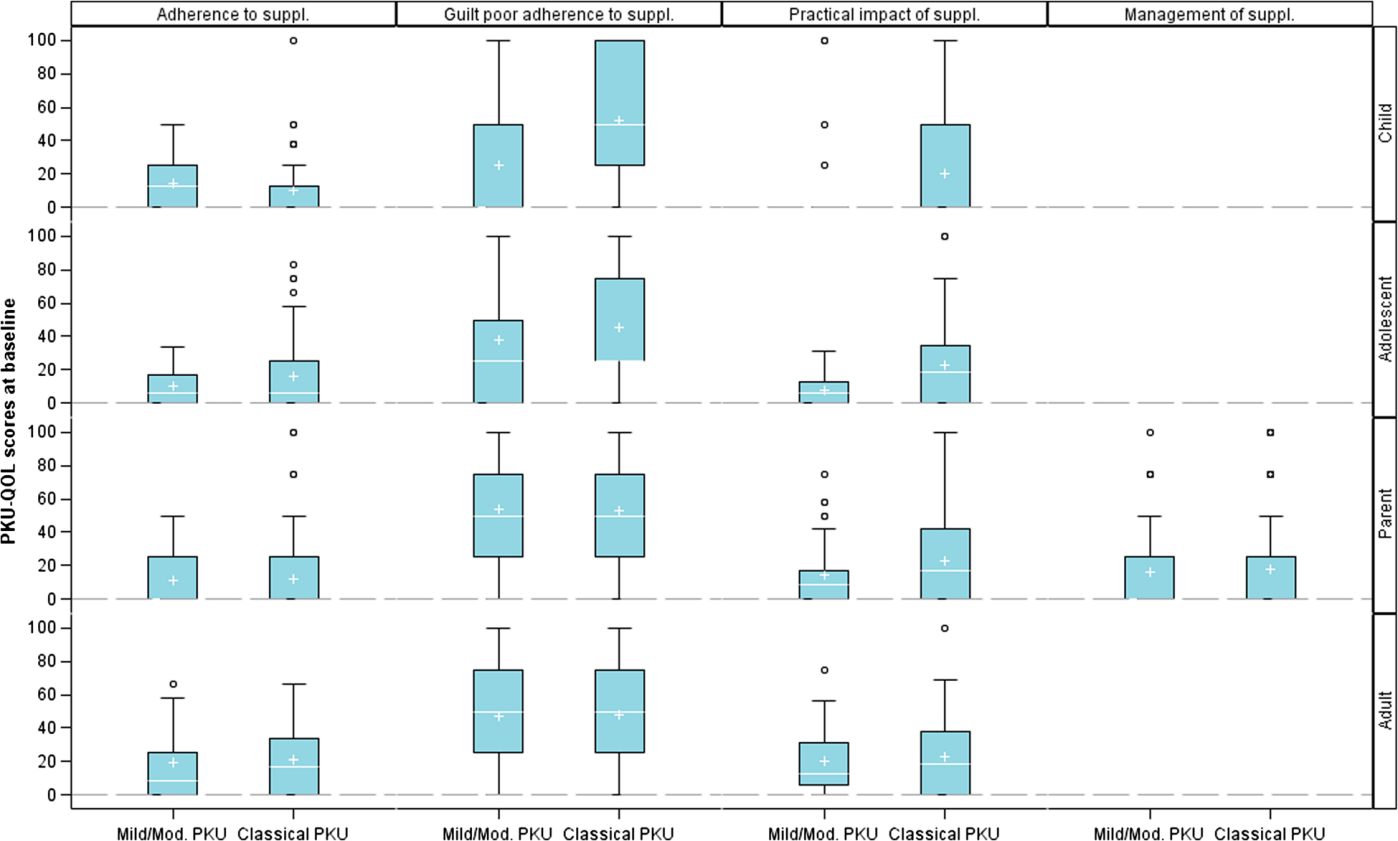
Comparison of PKU-QOL scores related to Phe-free amino acid supplements according to PKU severity (mild/moderate PKU: Phe level 600–1200 μmol/L; Classical PKU: Phe level >1200 μmol/L) in children (*N* = 92), adolescents (*N* = 110), parents (*N* = 253) and adults (*N* = 104). Box-plots presents the following: interquartile range (Q1-Q3); +: mean; –: median; bottom and top bars: observed minimum and maximum observed values; ○: outliers (i.e., values that are outside the distance of 1.5 times the interquartile range from Q1 or Q3)

All comparisons of PKU-QOL scores for subgroups based on the severity of PKU (as defined by blood Phe level at diagnosis) are reported in Additional file 1: Table S1. No clear association between the symptoms of PKU and the severity of PKU were observed. A consistent pattern emerged regarding the impact of Phe-free amino acid supplement intake according to the severity of PKU (Fig. 1): patients with classical PKU, in particular adolescents and children, reported a slightly greater impact of Phe-free amino acid supplements on practical and emotional (guilt related to poor adherence) domains.

An association was also found in adolescents between the impact of dietary restriction and the severity of PKU: adolescents with classical PKU reported a slightly poorer adherence to their diet and higher impact (social and emotional) (Additional file 1: Table S1). This finding was not replicated in adults. In children, the findings varied according to the respondents: the impact of the diet measured by the self-reported Child version did not differ according to the severity of PKU, whilst results from the Parent version showed children with classical PKU as having poorer dietary adherence and experiencing greater impact from the diet than children with mild or moderate PKU (Additional file 1: Table S1).

#### PKU-QOL scores according to overall health status

All comparisons related to the overall health status as assessed by the clinicians are reported in the Additional file 2: Table S2. Overall, patients with better health status according to the clinicians’ global rating tended to have lower symptom scores (i.e., less frequent symptoms) and lower PKU impact scores, particularly in Adult and Parent questionnaires. No patients’ health status was reported as “Poor” by their clinician, and very few as “Fair”; this reflects the overall good health status of patients with PKU, with very few having a health status rated as worse than “Good”.

No clear association was found between patients’ health status as assessed by the clinician and the impact of the Phe-free amino acid supplement administration or the dietary protein restriction scores.

#### PKU-QOL scores according to BH_4_ intake

**Fig. 2:**
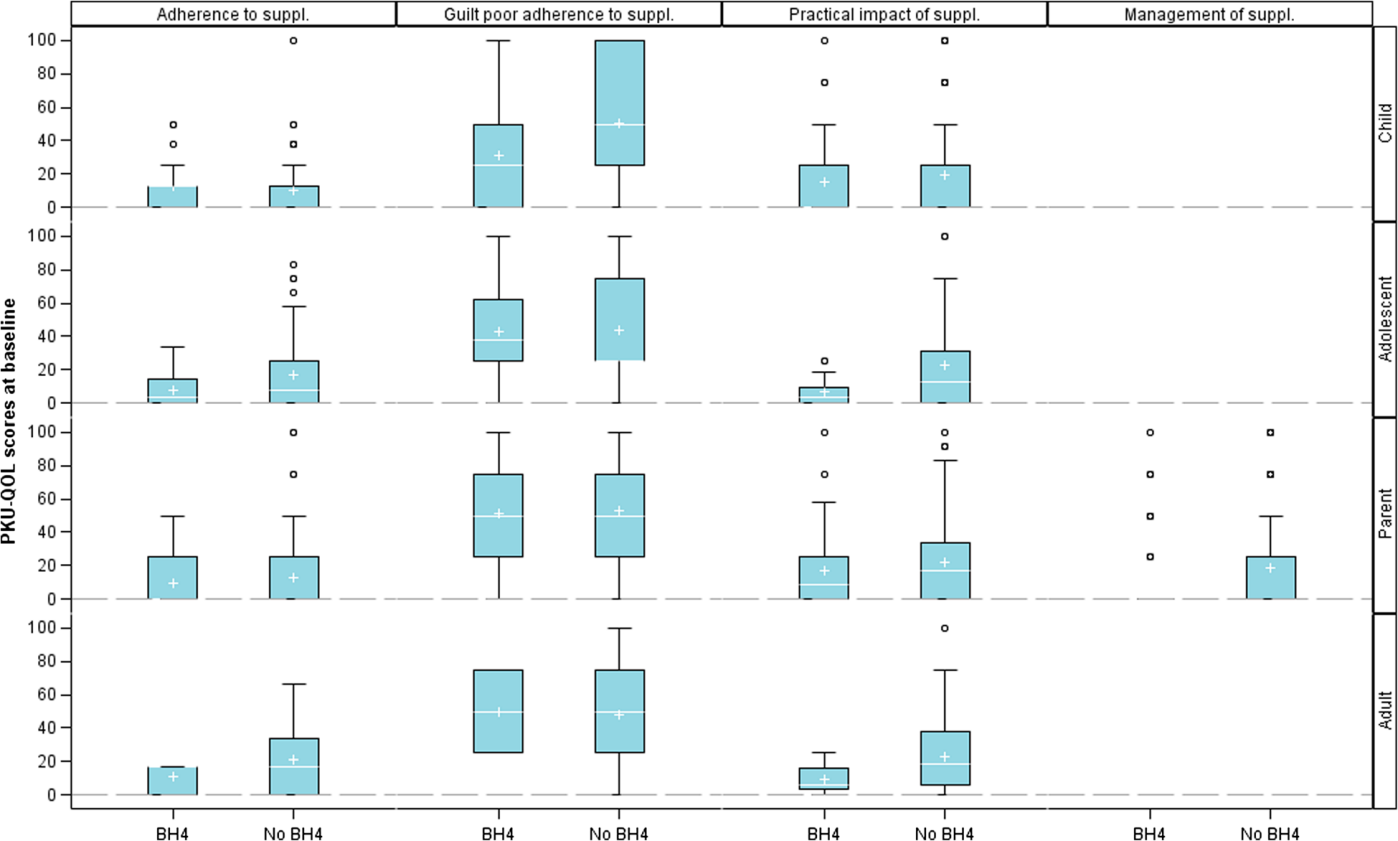
Comparison of PKU-QOL scores related to Phe-free amino acid supplements according to BH_4_ intake in children (*N* = 92), adolescents (*N* = 110), parents (*N* = 253) and adults (*N* = 104). Box-plots presents the following: interquartile range (Q1-Q3); +: mean; –: median; bottom and top bars: observed minimum and maximum observed values; ○: outliers (i.e., values that are outside the distance of 1.5 times the interquartile range from Q1 or Q3)

**Fig. 3:**
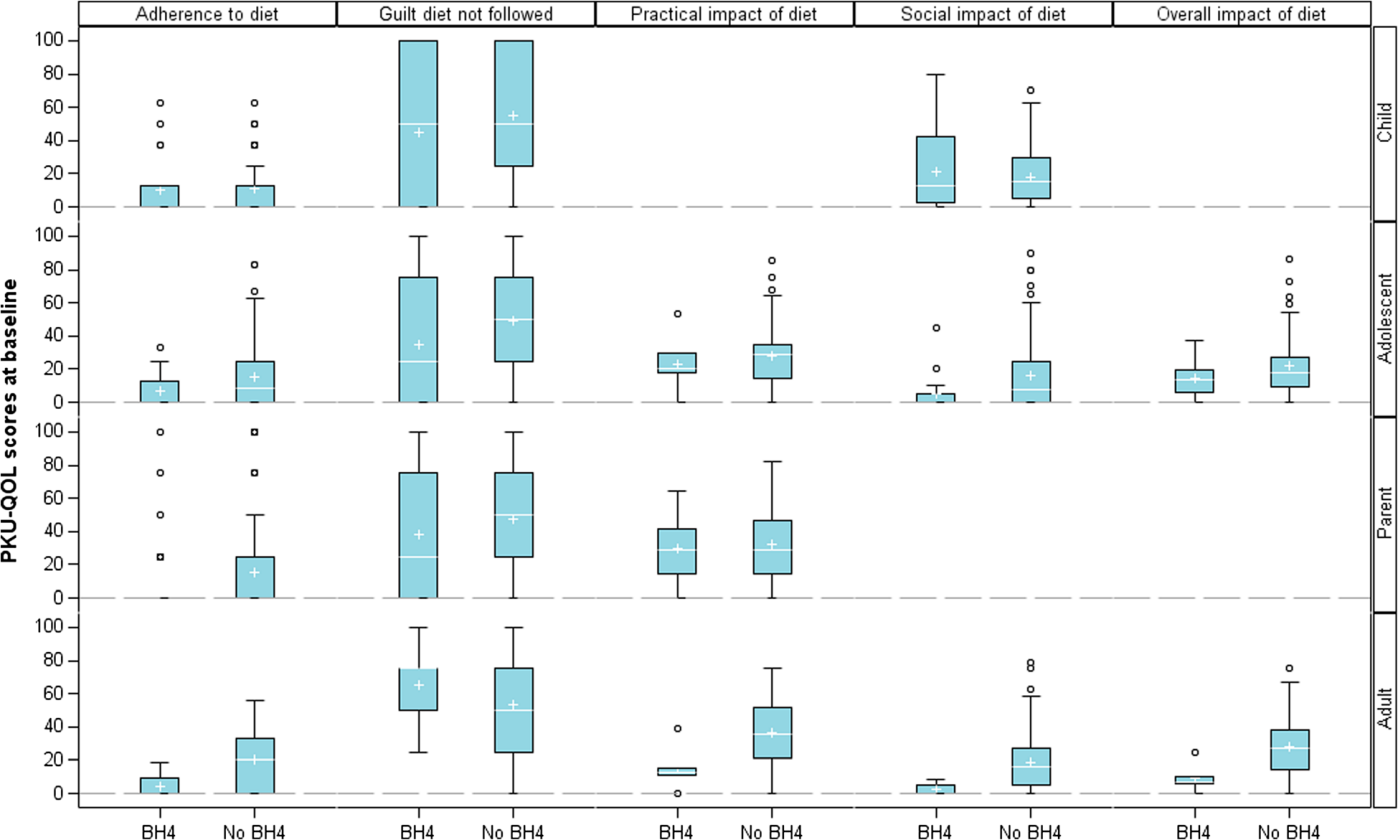
Comparison of PKU-QOL scores related to diet according to BH_4_ intake in children (*N* = 92), adolescents (*N* = 110), parents (*N* = 253) and adults (*N* = 104). Box-plots presents the following: interquartile range (Q1-Q3); +: mean; –: median; bottom and top bars: observed minimum and maximum observed values; ○: outliers (i.e., values that are outside the distance of 1.5 times the interquartile range from Q1 or Q3)

All BH_4_ intake-related comparisons are reported in Additional file 3: Table S3. Consistently for all groups the practical impact of Phe-free amino-acid supplement was lower in patients treated by BH_4_ than those with no BH_4_ intake who were treated with diet only, and for all PKU-QOLs except the Child version there was better adherence to Phe-free amino-acid supplement administration in patients treated by BH_4_ (Fig. 2 and Additional file 3: Table S3). The impact of the dietary restriction (overall impact as well as social and practical impact) was lower in adolescent and adult patients receiving pharmacological treatment with BH_4_ compared to patients not treated with BH_4_ (Fig. 3). Very little difference in PKU-QOL symptoms and PKU impact scores was observed between patients taking BH_4_ and those who did not.

## Discussion

This is the first study to investigate the HRQoL of a large international sample of patients with PKU using the PKU-QOL, a recently developed PKU-specific HRQoL questionnaire, as well as generic questionnaires. A total of 306 patients and 253 parents from seven countries participated in the study. This paper presents exploratory analyses performed post-hoc on data collected primarily for the purpose of validating the PKU-QOL, reflecting the secondary objective of describing HRQoL measured by instruments specifically designed to address issues characteristic of the PKU population. Because of the exploratory nature of this aspect of the research, caution has been used in interpreting the findings.

### HRQoL of PKU patients: generic questionnaires

All patients and parents completed age-specific generic HRQoL questionnaires (PedsQL, Child Health Questionnaire PF28, SF36). The children and adolescents reported PedsQL scores comparable to the US normative data sample, confirming previous study reports of consistently normal scores on generic questionnaires in patients with PKU [17–20]. On the SF36 questionnaire the adult group presented higher mean scores on the physical domains, but slightly lower mean scores on the mental domains compared to the US norm scores. Higher physical scores have been reported before by patients with PKU [22] and may be attributed to the fact that adult patients included in PKU studies are likely to be younger than adults in the reference group from the general populations – the mean age of adults recruited in this study was 25.8 years, compared with 50.7 years in the sample for the general population completing the SF-36 [36]. Alternatively this may also be because of a response shift: PKU patients grow up with the knowledge that poor adherence with dietary treatment may harm their health and abilities, and they may appreciate their own health more than the general population, possibly resulting in higher HRQoL scores [22]. Lower mental scores on generic questionnaires have been previously reported in PKU adults as well and may result from the lower mean IQ found in patients with PKU or from the negative effects of high blood Phe levels on executive function and mood [12–16, 22].

Parents reported scores on the CHQ-PF28 comparable to the US norms, except for a lower score on the emotional parental impact domain, indicating a higher emotional impact than the general population. Considering the severity of disease and burden of treatment this is an expected outcome, which confirms previous studies reporting parental fears of school failure [20] and normal overall parental QoL, but with an increased vulnerability of parents of younger children [38]. It has been demonstrated that family stress, perceived limited social support and loss of friendship are the most powerful predictors of lower HRQoL in parents of PKU patients [16, 38].

### HRQoL of PKU patients: PKU-specific questionnaire

The PKU-QOL addresses PKU specific issues such as PKU symptoms, impact of PKU and its management, dietary protein restriction and administration of Phe-free amino acid supplements. Patients of all ages gave highest scores to domains addressing the emotional impact of PKU and the management of PKU, including anxiety about high blood Phe levels and guilt related to poor adherence to dietary restrictions or supplement intake. The highest score in females in all patient groups was the score measuring anxiety regarding high blood Phe levels during pregnancy, which demonstrates that female patients are well aware of the risks to the foetus in an unplanned pregnancy [39].

Because the questionnaire addresses issues relevant only to PKU patients and their parents, it is not possible to compare scores to a control group from a general population. However, a comparison between patient groups was possible and allowed confirmation that these questionnaires are sensitive to HRQoL issues characteristic of the PKU populations, although further study will be necessary to confirm that they can demonstrate the effects of supportive interventions (such as referral to and support by a psychologist, improvement of knowledge through PKU camps, etc.), of treatment differences (such as BH_4_ versus diet only) and of future treatment tools.

#### Effects of severity of PKU

In PKU patients a major determinant for the burden of disease is disease severity, which determines tolerance to dietary Phe. Those patients with lower Phe tolerance necessarily require a more severe dietary protein restriction to maintain blood Phe within recommended ranges. The severity of protein restriction thus significantly affects adherence difficulty and quality of life. A commonly used severity classification for PKU is by blood Phe level at the time of diagnosis, with a Phe level of >1200 μmol/L classifying as severe (classical) PKU and a Phe level <1200 μmol/L as mild to moderate PKU. Due to the high overall scores on generic HRQoL questionnaires, previous studies could not demonstrate the clinically observed differences in HRQoL between these groups. Therefore, we evaluated differences in PKU-QOL scores between patients with mild-moderate and severe PKU. In this study more than two-thirds of the patients evaluated (68%) had classic PKU and this proportion was fairly stable across all age groups, although slightly higher in children (71%).

The differences in HRQoL between patients with mild-moderate and severe PKU observed in all age groups made sense from a clinical perspective. In the mild-moderate PKU group adolescents reported better health status and fewer mood swings, and adults and adolescents demonstrated a trend to lower emotional, practical, social and overall impact of PKU, compared to those with severe PKU. Also, adults demonstrated a trend to lower financial impact of PKU with less severe disease. Parents of mild-moderate patients reported lower practical, financial, and overall impact of PKU, and a trend for a lower emotional impact than parents of patients with severe PKU.

In the domain of dietary impact, adolescents with severe PKU reported higher overall impact of the diet (both social and practical impact), more guilt if the diet is not followed, less food enjoyment and trends for more food temptation than adolescents with mild-moderate disease. Parents of patients with severe PKU reported less food enjoyment and poorer adherence to diet, as well as trends for more difficulty with diet management, more practical impact and more guilt if the diet is not followed than parents with less severe PKU.

Remarkably these differences in the diet domain are not seen in the adult sample. This may result from higher accepted treatment blood Phe ranges in adults compared to children and adolescents in many countries, thus allowing a more relaxed diet restriction [1]. Furthermore, parents enforce adherence during childhood because they understand dietary treatment is important for good outcome, and fear possible long term effects of higher blood Phe. During adolescence acceptance of the importance of dietary therapy conflicts with fear of being different because of the diet, and becomes a major HRQoL issue.

This study clearly demonstrates that severity of PKU is negatively associated with the patients’ HRQoL and that treatment options enabling relaxation of diet may improve HRQoL as measured with a PKU-specific questionnaire. At this time the only pharmacological treatment for PKU is BH_4_ (combined with diet therapy) which may increase Phe tolerance in patients who are responsive. These patients may be able to considerably relax dietary Phe restrictions, include more natural protein in their diet, and/or decrease Phe-free amino acid supplement intake.

#### Effects of BH_4_ treatment

Previous studies evaluating the effects of BH_4_ treatment with generic HRQoL questionnaires were not able to demonstrate any of the striking beneficial effects clinically observed by professionals and patients [22, 40]. Therefore the cohort of patients receiving BH_4_ treatment (*n* = 69), in addition to dietary therapy and Phe-free amino acid supplement (except in very rare cases), was compared to the cohort of patients treated with diet and Phe-free amino acid supplement only (237). In the domain of PKU symptoms, adolescents treated with BH_4_ reported less sadness than patients not receiving BH_4_, while adults receiving BH_4_ presented a trend of less aggressiveness and less slow thinking. This could result from lower blood Phe values in patients treated with BH_4_, as higher blood Phe values are associated with mood disturbances and a lower sustained attention. Remarkably, parents of children treated with BH_4_ reported more mood swings. Compared to adolescents and adults treated with diet only, adolescents and adults receiving BH_4_ reported less food temptation and a lesser social impact of dietary restriction (feeling different because of diet, conflict with parents about diet, difficulty watching others eat, feeling socially restricted because of diet). In adults the overall impact of diet (social impact plus practical difficulties such as difficulty eating out, need to plan the meals in advance, time needed to prepare the meals) was lower in patients treated with BH_4_. Also, the domain “overall difficulty of the diet” (a single item score measuring the perceived difficulty of following the diet) presented lower scores in adolescents and a beneficial trend in adults receiving BH_4_. A lower financial impact was also reported by PKU adults receiving BH_4_.

Importantly, parents reported a better adherence to diet in children receiving BH_4_.

Children receiving BH_4_ treatment exhibited less guilt related to poor adherence to Phe-free supplement intake, while adolescents treated with BH_4_ reported a lower practical impact of Phe-free amino acid supplement administration. Adolescents, adults and parents reported a slightly better adherence to Phe-free amino acid supplement intake.

BH_4_ treatment is associated with better scores on the domains of the PKU-specific questionnaire in all ages and these results confirm the clinical observation that a less restricted diet and lower Phe-free amino acid supplement intake, often allowed by response to BH_4_, will positively affect the HRQoL of the patients. The higher score on mood swing in children using BH_4_ is as yet unexplained and future studies will need to address this finding.

The fact that the PKU-QOL questionnaires may allow description of differences in HRQoL between patients with differing severity of PKU, and patients receiving different treatment modalities confirms the usefulness of the instrument. The PKU-QOL is anticipated to become a valuable tool in the evaluation and care of patients with PKU, as well in understanding and documenting quality of life concerns specific to and characteristic of the PKU population.

### Limitations of the study

Patients and parents from seven countries participated in the study. Hence cross-cultural differences in HRQoL may exist, in particular due to differences in access to different treatment options. Differences in impact on HRQoL depending on treatment have been evaluated but, due to the small sample size in each country, these analyses could not be conducted at the country level. The scores on the generic questionnaires have been compared to the USA norm group, as norm scores were not available for the participating countries. This introduces a potential bias in terms of the matching of the demographics, culture and behaviour of the two groups. However, it was considered useful for the interpretation of results to have a reference, and as our cohort is the largest and internationally most diverse cohort of PKU patients ever evaluated for HRQoL, the US norm scores can be expected to be based on a comparable diversity. Furthermore, results clearly confirm previous findings in studies using national norm scores [17, 19, 20, 22, 40].

A larger sample size would have allowed more in-depth analysis; however the sample included was relatively large for this rare condition (we believe it was the largest cohort of PKU patients ever evaluated for HRQoL), and allowed the primary objective of the study – validation of the future usefulness of the instruments – to be achieved.

Finally, it is important to note that this analysis is exploratory by nature (it was performed to support a secondary objective of the study and involves a large number of statistical tests) and its results should be interpreted as such. In particular, all the findings resulting from the comparisons of PKU-QOL scores should be considered cautiously and would need to be confirmed in further studies. One of the main findings was the potential alleviation of the negative impact of diet restriction and Phe-free amino acid supplement intake in patients who were treated with BH_4_, which is suggested by our results. However, one of the questions related to this finding is whether this could be explained by a difference in PKU severity in patients treated with BH_4_. Patients with mild/moderate PKU are more likely to respond to BH_4_, which is confirmed in our sample (across all age groups, there were consistently more than 60% of the patients receiving BH_4_ who had mild/moderate PKU while they were fewer than 30% of patients not receiving BH_4_). So PKU severity may be a confounding factor in the association between PKU-QOL scores and BH_4_ intake; however, given the relatively small number of patients who were receiving BH_4_ in the sample, a subgroup analysis of this sample according to PKU severity was not possible. This important question should be further explored in future research.

### Use of the PKU-QOL

The PKU-QOL is now available in seven languages and has been demonstrated to be valid for use in France, Germany, Italy, The Netherlands, Spain, Turkey and the UK. It is a valid and reliable measure of the multifaceted impact of PKU on patients of different age groups (children, adolescents and adults) and their parents [24]. In the large cohort of patients and parents involved in the development and validation of the instrument, clinically observed differences were clearly and consistently reflected in the score differences between mild-moderate and severe patients; and in patients receiving different treatments, supporting the validity of the questionnaire. While the questionnaire was primarily developed to be used in the context of clinical research, it could also be a powerful tool in the context of patient care and follow-up.

In research, the PKU-QOL could be a valuable asset in studies evaluating interventions (such as educational programs and psychosocial referrals) as well as new treatment options, with repeated measurements over time.

In patient care, the questionnaire could be useful to provide the clinician with a standardized picture of their patient’s perspective. The use of questionnaires has been evaluated in many different chronic disorders and has been demonstrated to significantly increase dialogue on psychosocial function and emotional function in cohorts of adults and children with cancer, and to improve psychosocial outcome in children and adolescents with diabetes and adults with cancer [41–44]. An individual patient’s HRQoL can be followed over time, as well as before and after individual interventions (such as changes in diet or referrals to psychosocial professionals). In this case, the patient serves as his/her own control.

Values generated in this large sample and provided in this paper could be used as helpful references. However this does present a risk of bias, and cultural differences as well as treatment severity must be taken into account. For this reason, different reference scores are provided for mild-moderate and severe PKU patients. In particular, BH_4_-responsive patients who may tolerate less dietary restriction might be compared to the mild-moderate patients.

## Conclusions

Overall, patients with PKU showed good HRQoL, both with the generic and PKU-specific measures. Nonetheless, a negative impact of PKU on a patient’s life, in particular the emotional impact of PKU and its management, was delineated by the PKU-QOLs across all age groups. Patients with milder PKU and patients treated with BH_4_ reported a lower impact of dietary restriction and Phe-free amino acid supplement intake (social and practical), reflecting the relaxed constraints in these patients. These results support the ability of the PKU-QOL to identify HRQoL issues characteristic of the PKU population, and to measure HRQoL differences specific to severity of disease and treatment received. Further research is now needed to gain a better understanding of the effect of present and future PKU treatments on patient-centred outcomes, in particular in longitudinal studies that would allow the assessment of treatment effects over time.

### Intellectual property and condition of use

The PKU-QOLs are protected by international copyright – PKU-QOL © Merck Serono S.A. - Geneva – 2010-

The PKU-QOL is available freely for use in individual medical practice and in non-privately funded academic research. Access to the questionnaire, as well as further information on, or permission to use the PKU-QOL and/or its translations, can be found on http://www.proqolid.org

## Additional files

Additional file 1: Table S1. Comparisons of PKU-QOL scores according to the severity of PKU. This file includes four tables presenting in the child, adolescent, adult and parent samples the comparison of PKU-QOL scores according to the severity of PKU (mild-moderate PKU: Phe level 600–1200 μmol/L; Classical PKU: Phe level >1200 μmol/L). Additional file 2: Table S2. Comparisons of PKU-QOL scores according to the overall health status as assessed by the clinicians. This file includes four tables presenting in the child, adolescent, adult and parent samples the comparison of PKU-QOL scores according to the overall assessment of health status made by the clinicians (poor; fair; good; very good; excellent). Additional file 3: Table S3. Comparisons of PKU-QOL scores according to BH_4_ intake. This file includes four tables presenting in the child, adolescent, adult and parent samples the comparison of PKU-QOL scores according to BH_4_ intake (BH_4_ intake; no BH_4_ intake).

## Abbreviations

BH_4_: Tetrahydrobiopterin
CHQ-PF: Child Health Questionnaire–Parent Form
PedsQL: Pediatric Quality-of-Life Inventory
Phe: Phenylalanine
PKU: Phenylketonuria
PKU-QOL: PKU-specific Quality of Life questionnaire
HRQoL: Health-Related Quality of Life
SF-36: Medical Outcome Survey 36-item Short Form
