# Supplementary material for: Assessment of the impact of phenylketonuria and its treatment on quality of life of patients and parents from seven European countries
Source: Orphanet J Rare Dis. 2015 Jun 18;10:80. doi: 10.1186/s13023-015-0294-x (PMC4542123; doi:10.1186/s13023-015-0294-x)
Supplement: Additional file 1: Table S1. — Comparisons of PKU-QOL scores according to the severity of PKU. This file includes four tables presenting in the child, adolescent, adult and parent samples the comparison of PKU-QOL scores according to the severity of PKU (mild-moderate PKU: Phe level 600–1200 μmol/L; Classical PKU: Phe level >1200 μmol/L). [file 13023_2015_294_MOESM1_ESM.pdf]

Additional File 1 : Comparison of PKU-QOL scores according to severity of PKU (Mild-moderate PKU : Phe level 600-1200 µmol/L ; Classical PKU : Phe level >1200 µmol/L)

Table 1_a : Comparison of the Child PKU-QOL scores according to severity of PKU

Modules	Domains		Mild-moderate PKU (N=22)	Classical PKU (N=66)	p-value*
**Symptoms**	Headaches	**Mean (SD)**	18.8 (28.0)	14.5 (19.3)	0.817
		**Median (Q1 – Q3)**	0.0 (0.0 – 100.0)	0.0 (0.0 – 75.0)	
	Stomach aches	**Mean (SD)**	20.0 (25.1)	14.8 (21.7)	0.455
		**Median (Q1 – Q3)**	0.0 (0.0 – 75.0)	0.0 (0.0 – 100.0)	
	Tiredness	**Mean (SD)**	37.5 (32.9)	34.0 (24.9)	0.666
		**Median (Q1 – Q3)**	50.0 (0.0 – 100.0)	25.0 (0.0 – 100.0)	
	Irritability	**Mean (SD)**	25.0 (38.9)	18.0 (25.0)	0.948
		**Median (Q1 – Q3)**	0.0 (0.0 – 100.0)	0.0 (0.0 – 100.0)	
	Aggressiveness	**Mean (SD)**	20.0 (31.0)	10.2 (21.2)	0.238
		**Median (Q1 – Q3)**	0.0 (0.0 – 100.0)	0.0 (0.0 – 100.0)	
	Moodiness	**Mean (SD)**	22.5 (26.8)	15.6 (22.9)	0.334
		**Median (Q1 – Q3)**	0.0 (0.0 – 75.0)	0.0 (0.0 – 100.0)	
	Sadness	**Mean (SD)**	22.6 (29.5)	15.2 (21.2)	0.417
		**Median (Q1 – Q3)**	0.0 (0.0 – 100.0)	0.0 (0.0 – 100.0)	
	Anxiety	**Mean (SD)**	8.8 (18.6)	9.8 (18.7)	0.725
		**Median (Q1 – Q3)**	0.0 (0.0 – 50.0)	0.0 (0.0 – 75.0)	
	Lack of concentration	**Mean (SD)**	22.4 (29.9)	22.8 (28.5)	0.887
		**Median (Q1 – Q3)**	0.0 (0.0 – 100.0)	25.0 (0.0 – 100.0)	
	Slow thinking	**Mean (SD)**	22.4 (26.2)	23.2 (26.5)	0.938
		**Median (Q1 – Q3)**	25.0 (0.0 – 100.0)	25.0 (0.0 – 100.0)	
**PKU in general**	Emotional impact of PKU	**Mean (SD)**	35.3 (21.6)	32.1 (22.0)	0.496
		**Median (Q1 – Q3)**	33.3 (0.0 – 66.7)	25.0 (0.0 – 83.3)	
	Practical impact of PKU	**Mean (SD)**	16.1 (21.3)	10.0 (15.3)	0.325
		**Median (Q1 – Q3)**	0.0 (0.0 – 75.0)	0.0 (0.0 – 75.0)	
	Social impact of PKU	**Mean (SD)**	21.0 (21.0)	19.8 (17.3)	0.830
		**Median (Q1 – Q3)**	16.7 (0.0 – 91.7)	16.7 (0.0 – 75.0)	
	Overall impact of PKU	**Mean (SD)**	25.1 (17.1)	22.0 (14.9)	0.494
		**Median (Q1 – Q3)**	18.8 (0.0 – 71.9)	18.8 (0.0 – 78.1)	
	Anxiety – Blood test	**Mean (SD)**	8.3 (15.0)	13.7 (17.3)	0.181
		**Median (Q1 – Q3)**	0.0 (0.0 – 62.5)	12.5 (0.0 – 50.0)	
	Anxiety – Phe levels	**Mean (SD)**	58.3 (39.8)	42.6 (35.3)	0.102
		**Median (Q1 – Q3)**	50.0 (0.0 – 100.0)	25.0 (0.0 – 100.0)	

Table 1_a : Comparison of the Child PKU-QOL scores according to severity of PKU (cont'd)

Modules	Domains		Mild-moderate PKU (N=22)	Classical PKU (N=66)	p-value*
**Supplement administration**	Adherence to supplements	**Mean (SD)**	14.0 (17.6)	10.3 (17.6)	0.327
		**Median (Q1 – Q3)**	12.5 (0.0 – 50.0)	0.0 (0.0 – 100.0)	
	Practical impact of supplements	**Mean (SD)**	15.3 (33.4)	20.2 (31.7)	0.391
		**Median (Q1 – Q3)**	0.0 (0.0 – 100.0)	0.0 (0.0 – 100.0)	
	Guilt if poor adherence to supplements	**Mean (SD)**	25.0 (34.2)	52.0 (37.7)	**0.009**
		**Median (Q1 – Q3)**	0.0 (0.0 – 100.0)	50.0 (0.0 – 100.0)	
	Relationships within family because of supplements	**Mean (SD)**	19.4 (31.6)	6.7 (17.5)	0.090
		**Median (Q1 – Q3)**	0.0 (0.0 – 100.0)	0.0 (0.0 – 100.0)	
	Taste - Supplements	**Mean (SD)**	48.5 (25.7)	31.5 (31.0)	**0.027**
		**Median (Q1 – Q3)**	50.0 (0.0 – 100.0)	25.0 (0.0 – 100.0)	
**Dietary protein restriction**	Food temptations	**Mean (SD)**	40.1 (33.5)	26.8 (25.4)	0.129
		**Median (Q1 – Q3)**	37.5 (0.0 – 100.0)	25.0 (0.0 – 100.0)	
	Adherence to dietary protein restriction	**Mean (SD)**	9.9 (17.5)	10.7 (16.3)	0.761
		**Median (Q1 – Q3)**	0.0 (0.0 – 62.5)	0.0 (0.0 – 62.5)	
	Social impact of dietary protein restriction	**Mean (SD)**	26.1 (24.8)	16.8 (16.5)	0.196
		**Median (Q1 – Q3)**	15.0 (0.0 – 80.0)	12.5 (0.0 – 62.5)	
	Taste – low protein food	**Mean (SD)**	19.2 (20.8)	18.5 (24.4)	0.713
		**Median (Q1 – Q3)**	25.0 (0.0 – 50.0)	25.0 (0.0 – 100.0)	
	Food enjoyment	**Mean (SD)**	16.7 (22.8)	18.0 (24.2)	0.787
		**Median (Q1 – Q3)**	0.0 (0.0 – 50.0)	0.0 (0.0 – 100.0)	
	Guilt if dietary protein restriction not followed	**Mean (SD)**	38.2 (45.2)	57.9 (39.2)	0.059
		**Median (Q1 – Q3)**	0.0 (0.0 – 100.0)	62.5 (0.0 – 100.0)	
	Overall difficulty following dietary protein restriction	**Mean (SD)**	26.3 (37.7)	17.5 (24.4)	0.664
		**Median (Q1 – Q3)**	0.0 (0.0 – 100.0)	0.0 (0.0 – 100.0)	

**Non-parametric P-value for between-group comparisons: Mann-Whitney-Wilcoxon*
In bold p-value<0.05

Table 1_b : Comparison of the Adolescent PKU-QOL scores according to severity of PKU

Modules	Domains		Mild-moderate PKU (N=33)	Classical PKU (N=75)	p-value*
**Symptoms**	Self-rated status health	**Mean (SD)**	25.8 (26.2)	39.2 (22.5)	**0.012**
		**Median (Q1 – Q3)**	25.0 (0.0 – 75.0)	50.0 (0.0 – 75.0)	
	Headaches	**Mean (SD)**	15.3 (22.1)	21.9 (26.5)	0.245
		**Median (Q1 – Q3)**	0.0 (0.0 – 75.0)	25.0 (0.0 – 100.0)	
	Stomach aches	**Mean (SD)**	12.9 (18.1)	15.6 (22.4)	0.716
		**Median (Q1 – Q3)**	0.0 (0.0 – 50.0)	0.0 (0.0 – 100.0)	
	Tiredness	**Mean (SD)**	38.7 (26.5)	40.3 (25.4)	0.632
		**Median (Q1 – Q3)**	50.0 (0.0 – 100.0)	50.0 (0.0 – 100.0)	
	Irritability	**Mean (SD)**	18.9 (21.7)	26.7 (27.3)	0.213
		**Median (Q1 – Q3)**	25.0 (0.0 – 75.0)	25.0 (0.0 - 100.0)	
	Aggressiveness	**Mean (SD)**	9.1 (18.6)	12.5 (23.4)	0.494
		**Median (Q1 – Q3)**	0.0 (0.0 – 50.0)	0.0 (0.0 – 100.0)	
	Moodiness	**Mean (SD)**	12.9 (19.9)	22.9 (23.6)	**0.030**
		**Median (Q1 – Q3)**	0.0 (0.0 – 50.0)	25.0 (0.0 – 75.0)	
	Sadness	**Mean (SD)**	9.8 (18.7)	13.5 (20.1)	0.293
		**Median (Q1 – Q3)**	0.0 (0.0 – 50.0)	0.0 (0.0 – 75.0)	
	Anxiety	**Mean (SD)**	13.6 (23.5)	14.2 (24.0)	1.000
		**Median (Q1 – Q3)**	0.0 (0.0 – 100.0)	0.0 (0.0 – 100.0)	
	Lack of concentration	**Mean (SD)**	16.7 (27.7)	21.2 (22.9)	0.140
		**Median (Q1 – Q3)**	0.0 (0.0 – 100.0)	25.0 (0.0 – 75.0)	
	Slow thinking	**Mean (SD)**	9.8 (16.5)	16.0 (21.1)	0.171
		**Median (Q1 – Q3)**	0.0 (0.0 – 50.0)	0.0 (0.0 – 75.0)	
**PKU in general**	Emotional impact of PKU	**Mean (SD)**	26.1 (14.9)	34.9 (21.5)	0.115
		**Median (Q1 – Q3)**	27.5 (0.0 – 60.0)	30.0 (0.0 – 90.0)	
	Practical impact of PKU	**Mean (SD)**	9.7 (13.2)	12.7 (14.1)	0.440
		**Median (Q1 – Q3)**	4.2 (0.0 – 33.3)	8.3 (0.0 – 50.0)	
	Social impact of PKU	**Mean (SD)**	11.6 (10.7)	17.0 (15.5)	0.106
		**Median (Q1 – Q3)**	8.3 (0.0 – 33.3)	16.7 (0.0 – 91.7)	
	Overall impact of PKU	**Mean (SD)**	17.8 (9.3)	25.0 (16.9)	0.067
		**Median (Q1 – Q3)**	17.5 (0.0 – 34.1)	22.6 (0.0 – 85.0)	
	Anxiety - Blood test	**Mean (SD)**	10.2 (21.6)	11.5 (20.5)	0.690
		**Median (Q1 – Q3)**	0.0 (0.0 – 100.0)	0.0 (0.0 – 87.5)	
	Anxiety - Phe levels	**Mean (SD)**	30.5 (30.3)	38.2 (32.2)	0.255
		**Median (Q1 – Q3)**	25.0 (0.0 – 100.0)	25.0 (0.0 – 100.0)	

Table 1_b : Comparison of the Adolescent PKU-QOL scores according to severity of PKU (cont'd)

Modules	Domains		Mild-moderate PKU (N=33)	Classical PKU (N=75)	p-value*
**Supplement administration**	Adherence to supplements	**Mean (SD)**	10.3 (11.0)	16.1 (20.9)	0.590
		**Median (Q1 – Q3)**	6.3 (0.0 – 33.3)	6.3 (0.0 – 83.3)	
	Practical impact of supplements	**Mean (SD)**	7.4 (8.5)	22.8 (25.5)	**0.013**
		**Median (Q1 – Q3)**	6.3 (0.0 – 31.3)	18.8 (0.0 – 100.0)	
	Guilt if poor adherence to supplements	**Mean (SD)**	37.5 (34.3)	45.4 (36.4)	0.396
		**Median (Q1 – Q3)**	25.0 (0.0 – 100.0)	25.0 (0.0 – 100.0)	
	Relationships within family because of supplements	**Mean (SD)**	14.3 (21.8)	14.6 (26.5)	0.605
		**Median (Q1 – Q3)**	0.0 (0.0 – 75.0)	0.0 (0.0 – 100.0)	
	Taste - Supplements	**Mean (SD)**	46.4 (21.3)	38.9 (25.5)	0.065
		**Median (Q1 – Q3)**	50.0 (0.0 – 75.0)	37.5 (0.0 – 100.0)	
**Dietary protein restriction**	Food temptations	**Mean (SD)**	18.8 (26.7)	28.6 (28.4)	0.106
		**Median (Q1 – Q3)**	0.0 (0.0 – 100.0)	25.0 (0.0 – 100.0)	
	Adherence to dietary protein restriction	**Mean (SD)**	7.5 (10.8)	15.3 (20.0)	0.115
		**Median (Q1 – Q3)**	0.0 (0.0 – 33.3)	10.4 (0.0 – 83.3)	
	Practical impact of dietary protein restriction	**Mean (SD)**	20.2 (9.7)	29.0 (19.3)	0.188
		**Median (Q1 – Q3)**	20.7 (3.6 – 30.0)	28.6 (0.0 – 85.7)	
	Social impact of dietary protein restriction	**Mean (SD)**	5.5 (9.6)	16.3 (22.2)	0.064
		**Median (Q1 – Q3)**	2.5 (0.0 – 40.0)	7.5 (0.0 – 90.0)	
	Overall impact of dietary protein restriction	**Mean (SD)**	12.1 (8.8)	22.9 (18.2)	**0.018**
		**Median (Q1 – Q3)**	13.1 (0.0 – 31.3)	18.2 (0.0 – 86.4)	
	Taste – low protein food	**Mean (SD)**	21.4 (19.3)	26.8 (26.9)	0.680
		**Median (Q1 – Q3)**	25.0 (0.0 – 50.0)	25.0 (0.0 – 100.0)	
	Food enjoyment	**Mean (SD)**	7.9 (20.5)	18.6 (27.8)	**0.048**
		**Median (Q1 – Q3)**	0.0 (0.0 – 75.0)	0.0 (0.0 – 100.0)	
	Guilt if dietary protein restriction not followed	**Mean (SD)**	31.8 (36.3)	51.4 (34.3)	**0.022**
		**Median (Q1 – Q3)**	25.0 (0.0 – 100.0)	50.0 (0.0 – 100.0)	
	Overall difficulty following dietary protein restriction	**Mean (SD)**	7.1 (14.0)	20.1 (28.7)	0.052
		**Median (Q1 – Q3)**	0.0 (0.0 – 50.0)	0.0 (0.0 – 100.0)	

**Non-parametric P-value for between-group comparisons: Mann-Whitney-Wilcoxon*
In bold p-value<0.05

Table 1_c : Comparison of the Adult PKU-QOL scores according to severity of PKU					
**Modules**	**Domains**		**Mild-moderate PKU (N=37)**	**Classical PKU (N=67)**	**p-value***
**Symptoms**	Self-rated health status	**Mean (SD)**	35.4 (22.7)	32.5 (23.0)	0.370
		**Median (Q1 – Q3)**	50.0 (0.0 – 75.0)	25.0 (0.0 – 100.0)	
	Headaches	**Mean (SD)**	23.6 (23.9)	20.1 (23.7)	0.426
		**Median (Q1 – Q3)**	25.0 (0.0 – 75.0)	12.5 (0.0 – 75.0)	
	Stomach aches	**Mean (SD)**	18.8 (25.6)	15.5 (25.9)	0.397
		**Median (Q1 – Q3)**	0.0 (0.0 – 75.0)	0.0 (0.0 – 100.0)	
	Tiredness	**Mean (SD)**	44.4 (26.8)	44.3 (28.0)	0.945
		**Median (Q1 – Q3)**	50.0 (0.0 – 100.0)	50.0 (0.0 – 100.0)	
	Trembling hands	**Mean (SD)**	11.8 (21.1)	12.9 (24.1)	0.989
		**Median (Q1 – Q3)**	0.0 (0.0 – 75.0)	0.0 (0.0 – 100.0)	
	Irritability	**Mean (SD)**	32.6 (26.6)	34.0 (24.1)	0.752
		**Median (Q1 – Q3)**	37.5 (0.0 – 100.0)	25.0 (0.0 – 75.0)	
	Aggressiveness	**Mean (SD)**	13.2 (21.1)	12.1 (21.6)	0.688
		**Median (Q1 – Q3)**	0.0 (0.0 – 75.0)	0.0 (0.0 – 75.0)	
	Moodiness	**Mean (SD)**	22.2 (23.0)	23.5 (25.9)	0.919
		**Median (Q1 – Q3)**	25.0 (0.0 – 50.0)	25.0 (0.0 – 75.0)	
	Sadness	**Mean (SD)**	30.6 (29.9)	28.0 (27.6)	0.733
		**Median (Q1 – Q3)**	25.0 (0.0 – 100.0)	25.0 (0.0 – 100.0)	
	Anxiety	**Mean (SD)**	27.1 (30.7)	19.3 (28.4)	0.180
		**Median (Q1 – Q3)**	25.0 (0.0 – 100.0)	0.0 (0.0 – 100.0)	
	Lack of concentration	**Mean (SD)**	25.7 (27.7)	23.9 (25.4)	0.823
		**Median (Q1 – Q3)**	25.0 (0.0 – 100.0)	25.0 (0.0 – 100.0)	
	Slow thinking	**Mean (SD)**	13.9 (22.7)	20.5 (28.4)	0.268
		**Median (Q1 – Q3)**	0.0 (0.0 – 75.0)	0.0 (0.0 – 100.0)	
**PKU in general**	Emotional impact of PKU	**Mean (SD)**	43.1 (21.6)	45.4 (22.5)	0.586
		**Median (Q1 – Q3)**	45.0 (0.0 – 95.0)	45.0 (0.0 – 100.0)	
	Practical impact of PKU	**Mean (SD)**	16.0 (17.0)	18.9 (16.9)	0.321
		**Median (Q1 – Q3)**	8.3 (0.0 – 58.3)	16.7 (0.0 – 75.0)	
	Social impact of PKU	**Mean (SD)**	15.2 (14.1)	19.2 (16.0)	0.299
		**Median (Q1 – Q3)**	12.5 (0.0 – 68.8)	16.7 (0.0 – 66.7)	
	Overall impact of PKU	**Mean (SD)**	27.0 (13.1)	31.5 (16.3)	0.379
		**Median (Q1 – Q3)**	25.0 (8.3 – 54.2)	28.1 (2.1 – 68.8)	
	Anxiety - Blood test	**Mean (SD)**	12.9 (23.1)	9.4 (21.7)	0.211
		**Median (Q1 – Q3)**	0.0 (0.0 – 100.0)	0.0 (0.0 – 100.0)	
	Anxiety - Phe levels	**Mean (SD)**	36.8 (25.0)	36.6 (28.6)	0.914
		**Median (Q1 – Q3)**	25.0 (0.0 – 100.0)	25.0 (0.0 – 100.0)	
	Anxiety - Phe levels during pregnancy	**Mean (SD)**	77.2 (23.7)	82.7 (25.0)	0.257
		**Median (Q1 – Q3)**	75.0 (25.0 – 100.0)	100.0 (0.0 – 100.0)	
	Financial impact of PKU	**Mean (SD)**	9.7 (17.2)	23.9 (30.3)	**0.017**
		**Median (Q1 – Q3)**	0.0 (0.0 – 75.0)	25.0 (0.0 – 100.0)	
	Information on PKU	**Mean (SD)**	34.0 (20.0)	34.3 (26.4)	0.777
		**Median (Q1 – Q3)**	25.0 (0.0 – 75.0)	25.0 (0.0 – 100.0)	

**Table 1_c : Comparison of the Adult PKU-QOL scores according to severity of PKU (cont'd)**					
**Modules**	**Domains**		**Mild-moderate PKU (N=37)**	**Classical PKU (N=67)**	**p-value***
**Supplement administration**	Adherence to supplements	**Mean (SD)**	19.1 (21.8)	20.8 (19.8)	0.632
		**Median (Q1 – Q3)**	8.3 (0.0 – 66.7)	16.7 (0.0 – 66.7)	
	Practical impact of supplements	**Mean (SD)**	20.4 (20.3)	22.4 (21.9)	0.837
		**Median (Q1 – Q3)**	12.5 (0.0 – 75.0)	18.8 (0.0 – 100.0)	
	Guilt if poor adherence to supplements	**Mean (SD)**	47.4 (32.2)	48.0 (33.4)	0.978
		**Median (Q1 – Q3)**	50.0 (0.0 – 100.0)	50.0 (0.0 – 100.0)	
	Relationships within family because of supplements	**Mean (SD)**	6.6 (18.3)	12.5 (23.1)	0.234
		**Median (Q1 – Q3)**	0.0 (0.0 – 75.0)	0.0 (0.0 – 100.0)	
	Taste - Supplements	**Mean (SD)**	50.0 (23.6)	49.6 (26.2)	0.892
		**Median (Q1 – Q3)**	50.0 (25.0 – 100.0)	50.0 (0.0 – 100.0)	
**Dietary protein restriction**	Food temptations	**Mean (SD)**	33.9 (22.0)	35.9 (26.1)	0.526
		**Median (Q1 – Q3)**	37.5 (0.0 – 100.0)	50.0 (0.0 – 100.0)	
	Adherence to dietary protein restriction	**Mean (SD)**	14.6 (14.7)	21.7 (19.0)	0.150
		**Median (Q1 – Q3)**	15.0 (0.0 – 43.8)	20.0 (0.0 – 56.3)	
	Social impact of dietary protein-restriction	**Mean (SD)**	15.7 (17.3)	18.9 (19.0)	0.360
		**Median (Q1 – Q3)**	9.2 (0.0 – 58.3)	12.5 (0.0 – 79.2)	
	Practical impact of dietary protein restriction	**Mean (SD)**	35.3 (22.0)	35.0 (19.3)	0.971
		**Median (Q1 – Q3)**	32.1 (0.0 – 75.0)	35.7 (0.0 – 71.4)	
	Overall impact of dietary protein restriction	**Mean (SD)**	26.5 (18.0)	26.6 (17.2)	0.987
		**Median (Q1 – Q3)**	26.1 (0.0 – 67.3)	25.0 (0.0 – 75.0)	
	Taste – low protein food	**Mean (SD)**	35.9 (15.7)	32.5 (22.8)	0.409
		**Median (Q1 – Q3)**	25.0 (25.0 – 75.0)	25.0 (0.0 – 100.0)	
	Food enjoyment	**Mean (SD)**	22.9 (29.4)	27.5 (29.7)	0.483
		**Median (Q1 – Q3)**	25.0 (0.0 – 100.0)	25.0 (0.0 – 100.0)	
	Guilt if dietary protein restriction not followed	**Mean (SD)**	55.4 (30.1)	53.3 (32.4)	0.797
		**Median (Q1 – Q3)**	50.0 (0.0 – 100.0)	50.0 (0.0 – 100.0)	
	Overall difficulty following dietary protein restriction	**Mean (SD)**	30.2 (30.4)	27.5 (27.3)	0.771
		**Median (Q1 – Q3)**	25.0 (0.0 – 100.0)	25.0 (0.0 – 100.0)	

**Non-parametric P-value for between-group comparisons: Mann-Whitney-Wilcoxon*
In bold p-value<0.05

Table 1_d : Comparison of the Parent PKU-QOL scores according to patient's severity of PKU

Modules	Domains		Mild-moderate PKU (N=75)	Classical PKU (N=172)	p-value*
**Symptoms**	Child health status	**Mean (SD)**	25.0 (21.4)	34.1 (24.8)	**0.010**
		**Median (Q1 – Q3)**	25.0 (0.0 – 75.0)	25.0 (0.0 – 100.0)	
	Headaches	**Mean (SD)**	12.0 (19.9)	15.3 (21.1)	0.201
		**Median (Q1 – Q3)**	0.0 (0.0 – 75.0)	0.0 (0.0 – 75.0)	
	Stomach aches	**Mean (SD)**	21.0 (26.6)	17.7 (23.3)	0.482
		**Median (Q1 – Q3)**	0.0 (0.0 – 100.0)	0.0 (0.0 – 75.0)	
	Tiredness	**Mean (SD)**	31.7 (26.4)	31.9 (24.5)	0.753
		**Median (Q1 – Q3)**	25.0 (0.0 – 100.0)	25.0 (0.0 – 100.0)	
	Irritability	**Mean (SD)**	31.0 (26.3)	38.5 (27.8)	0.053
		**Median (Q1 – Q3)**	25.0 (0.0 – 100.0)	50.0 (0.0 – 100.0)	
	Aggressiveness	**Mean (SD)**	10.6 (17.6)	14.0 (23.5)	0.663
		**Median (Q1 – Q3)**	0.0 (0.0 – 75.0)	0.0 (0.0 – 100.0)	
	Moodiness	**Mean (SD)**	27.7 (25.7)	27.9 (25.7)	0.988
		**Median (Q1 – Q3)**	25.0 (0.0 – 100.0)	25.0 (0.0 – 100.0)	
	Sadness	**Mean (SD)**	16.1 (20.6)	19.0 (21.5)	0.287
		**Median (Q1 – Q3)**	0.0 (0.0 – 75.0)	25.0 (0.0 – 100.0)	
	Anxiety	**Mean (SD)**	12.7 (20.0)	14.5 (22.3)	0.634
		**Median (Q1 – Q3)**	0.0 (0.0 – 75.0)	0.0 (0.0 – 100.0)	
	Lack of concentration	**Mean (SD)**	29.1 (29.3)	30.2 (27.3)	0.634
		**Median (Q1 – Q3)**	25.0 (0.0 – 100.0)	25.0 (0.0 – 100.0)	
	Slow thinking	**Mean (SD)**	17.1 (25.0)	20.4 (26.7)	0.337
		**Median (Q1 – Q3)**	0.0 (0.0 – 100.0)	0.0 (0.0 – 100.0)	
**PKU in general**	Emotional impact of PKU	**Mean (SD)**	37.5 (21.3)	44.9 (23.2)	**0.023**
		**Median (Q1 – Q3)**	34.4 (0.0 – 87.5)	43.8 (0.0 – 100.0)	
	Practical impact of PKU	**Mean (SD)**	10.0 (12.1)	16.3 (15.2)	**0.003**
		**Median (Q1 – Q3)**	5.0 (0.0 – 41.7)	12.5 (0.0 – 70.0)	
	Social impact of PKU	**Mean (SD)**	13.8 (12.3)	18.5 (18.1)	0.163
		**Median (Q1 – Q3)**	10.0 (0.0 – 50.0)	15.0 (0.0 – 90.0)	
	Overall impact of PKU	**Mean (SD)**	19.2 (12.5)	25.9 (16.2)	**0.004**
		**Median (Q1 – Q3)**	18.3 (0.0 – 50.0)	23.2 (0.0 – 76.9)	
	Child anxiety - Blood test	**Mean (SD)**	20.4 (27.9)	25.0 (27.9)	0.144
		**Median (Q1 – Q3)**	12.5 (0.0 – 100.0)	12.5 (0.0 – 100.0)	
	Impact of child anxiety - Blood test	**Mean (SD)**	23.1 (29.5)	25.1 (28.3)	0.431
		**Median (Q1 – Q3)**	12.5 (0.0 – 100.0)	12.5 (0.0 – 100.0)	
	Anxiety - Phe levels	**Mean (SD)**	52.4 (31.0)	56.3 (32.6)	0.352
		**Median (Q1 – Q3)**	50.0 (0.0 – 100.0)	50.0 (0.0 – 100.0)	
	Financial impact of PKU	**Mean (SD)**	17.8 (24.5)	30.0 (30.3)	**0.003**
		**Median (Q1 – Q3)**	0.0 (0.0 – 100.0)	25.0 (0.0 – 100.0)	
	Information on PKU	**Mean (SD)**	29.1 (21.1)	32.5 (26.0)	0.391
		**Median (Q1 – Q3)**	25.0 (0.0 – 100.0)	25.0 (0.0 – 100.0)	

Table 1_d : Comparison of the Parent PKU-QOL scores according to patient's severity of PKU (cont'd)					
**Modules**	**Domains**		**Mild-moderate PKU (N=75)**	**Classical PKU (N=172)**	**p-value***
**Supplement administration**	Adherence to supplements	**Mean (SD)**	10.7 (16.1)	11.8 (18.9)	0.871
		**Median (Q1 – Q3)**	0.0 (0.0 – 50.0)	0.0 (0.0 – 100.0)	
	Management of supplements	**Mean (SD)**	16.3 (27.3)	18.0 (27.7)	0.605
		**Median (Q1 – Q3)**	0.0 (0.0 – 100.0)	0.0 (0.0 – 100.0)	
	Practical impact of supplements	**Mean (SD)**	14.5 (19.0)	22.5 (24.4)	**0.041**
		**Median (Q1 – Q3)**	8.3 (0.0 – 75.0)	16.7 (0.0 – 100.0)	
	Guilt if poor adherence to supplements	**Mean (SD)**	54.0 (30.5)	52.7 (35.4)	0.863
		**Median (Q1 – Q3)**	50.0 (0.0 – 100.0)	50.0 (0.0 – 100.0)	
	Relationships within family because of supplements	**Mean (SD)**	19.9 (26.0)	18.9 (26.4)	0.739
		**Median (Q1 – Q3)**	0.0 (0.0 – 75.0)	0.0 (0.0 – 100.0)	
**Dietary protein restriction**	Adherence to dietary protein restriction	**Mean (SD)**	8.7 (20.9)	14.9 (25.6)	0.098
		**Median (Q1 – Q3)**	0.0 (0.0 – 100.0)	0.0 (0.0 – 100.0)	
	Management of dietary protein restriction	**Mean (SD)**	19.7 (18.2)	26.4 (23.0)	0.099
		**Median (Q1 – Q3)**	16.7 (0.0 – 70.8)	20.8 (0.0 – 100.0)	
	Practical impact of dietary protein restriction	**Mean (SD)**	26.7 (16.3)	33.7 (21.7)	0.070
		**Median (Q1 – Q3)**	28.6 (0.0 – 64.3)	32.7 (0.0 – 82.1)	
	Child food enjoyment	**Mean (SD)**	11.2 (17.0)	23.0 (25.7)	**0.003**
		**Median (Q1 – Q3)**	0.0 (0.0 – 75.0)	25.0 (0.0 – 100.0)	
	Guilt if dietary protein restriction not followed	**Mean (SD)**	41.2 (30.4)	46.7 (37.0)	0.431
		**Median (Q1 – Q3)**	25.0 (0.0 – 100.0)	50.0 (0.0 – 100.0)	

**Non-parametric P-value for between-group comparisons: Mann-Whitney-Wilcoxon*
In bold p-value<0.05
