# Supplementary material for: Assessment of the impact of phenylketonuria and its treatment on quality of life of patients and parents from seven European countries
Source: Orphanet J Rare Dis. 2015 Jun 18;10:80. doi: 10.1186/s13023-015-0294-x (PMC4542123; doi:10.1186/s13023-015-0294-x)
Supplement: Additional file 2: Table S2. — Comparisons of PKU-QOL scores according to the overall health status as assessed by the clinicians. This file includes four tables presenting in the child, adolescent, adult and parent samples the comparison of PKU-QOL scores according to the overall assessment of health status made by the clinicians (poor; fair; good; very good; excellent). [file 13023_2015_294_MOESM2_ESM.pdf]

Additional File 2 : Comparison of PKU-QOL scores according to overall assessment of health status

Table 2_a : Comparison of the Child PKU-QOL scores according to overall assessment of health status

Modules	Domains		Poor (N=0)	Fair (N=2)	Good (N=19)	Very good (N=41)	Excellent (N=28)	p-value*
Symptoms	Headaches	Mean (SD)	na	12.5 (17.7)	16.7 (17.1)	13.1 (18.8)	19.2 (28.6)	0.829
		Median (Q1 – Q3)	na	12.5 (0.0 – 25.0)	25.0 (0.0 – 50.0)	0.0 (0.0 – 50.0)	0.0 (0.0 – 100.0)	
	Stomach aches	Mean (SD)	na	50.0 (70.7)	15.3 (21.2)	17.5 (22.1)	10.6 (17.6)	0.518
		Median (Q1 – Q3)	na	50.0 (0.0 – 100.0)	0.0 (0.0 – 75.0)	0.0 (0.0 – 75.0)	0.0 (0.0 – 50.0)	
	Tiredness	Mean (SD)	na	62.5 (53.0)	38.9 (24.6)	30.6 (26.2)	36.5 (26.7)	0.382
		Median (Q1 – Q3)	na	62.5 (25.0 – 100.0)	50.0 (0.0 – 75.0)	25.0 (0.0 – 100.0)	50.0 (0.0 – 100.0)	
	Irritability	Mean (SD)	na	50.0 (70.7)	16.7 (24.3)	21.3 (29.7)	15.4 (26.5)	0.738
		Median (Q1 – Q3)	na	50.0 (0.0 – 100.0)	0.0 (0.0 – 75.0)	0.0 (0.0 – 100.0)	0.0 (0.0 – 100.0)	
	Aggressiveness	Mean (SD)	na	50.0 (70.7)	18.1 (30.7)	11.3 (20.4)	7.7 (17.0)	0.534
		Median (Q1 – Q3)	na	50.0 (0.0 – 100.0)	0.0 (0.0 – 100.0)	0.0 (0.0 – 75.0)	0.0 (0.0 – 75.0)	
	Moodiness	Mean (SD)	na	50.0 (70.7)	11.1 (19.6)	21.3 (24.4)	11.5 (19.0)	0.205
		Median (Q1 – Q3)	na	50.0 (0.0 – 100.0)	0.0 (0.0 – 50.0)	12.5 (0.0 – 75.0)	0.0 (0.0 – 50.0)	
	Sadness	Mean (SD)	na	62.5 (53.0)	9.7 (15.2)	18.8 (25.8)	14.8 (18.7)	0.180
		Median (Q1 – Q3)	na	62.5 (25.0 - 100.0)	0.0 (0.0 – 50.0)	0.0 (0.0 – 100.0)	0.0 (0.0 – 50.0)	
	Anxiety	Mean (SD)	na	25.0 (35.4)	5.6 (13.7)	11.9 (20.4)	6.7 (16.7)	0.347
		Median (Q1 – Q3)	na	25.0 (0.0 – 50.0)	0.0 (0.0 – 50.0)	0.0 (0.0 – 75.0)	0.0 (0.0 – 50.0)	
	Lack of concentration	Mean (SD)	na	50.0 (35.4)	20.6 (28.3)	21.3 (26.2)	23.0 (31.4)	0.539
		Median (Q1 – Q3)	na	50.0 (25.0 – 75.0)	25.0 (0.0 – 100.0)	0.0 (0.0 – 75.0)	0.0 (0.0 – 100.0)	
	Slow thinking	Mean (SD)	na	37.5 (17.7)	26.5 (22.5)	23.5 (31.2)	19.0 (22.0)	0.453
		Median (Q1 – Q3)	na	37.5 (25.0 – 50.0)	25.0 (0.0 – 75.0)	0.0 (0.0 – 100.0)	25.0 (0.0 – 75.0)	
PKU in general	Emotional impact of PKU	Mean (SD)	na	41.7 (23.6)	38.0 (24.5)	32.7 (23.3)	30.1 (18.0)	0.690
		Median (Q1 – Q3)	na	41.7 (25.0 – 58.3)	37.5 (0.0 – 75.0)	29.2 (0.0 – 83.3)	29.2 (0.0 – 66.7)	
	Practical impact of PKU	Mean (SD)	na	25.0 (35.4)	9.0 (12.7)	11.3 (18.5)	11.6 (16.2)	0.900
		Median (Q1 – Q3)	na	25.0 (0.0 – 50.0)	0.0 (0.0 – 37.5)	0.0 (0.0 – 75.0)	0.0 (0.0 – 50.0)	
	Social impact of PKU	Mean (SD)	na	37.5 (29.5)	23.6 (12.9)	21.5 (21.5)	13.5 (12.5)	0.086
		Median (Q1 – Q3)	na	37.5 (16.7 – 58.3)	25.0 (0.0 – 50.0)	16.7 (0.0 – 91.7)	8.3 (0.0 – 33.3)	
	Overall impact of PKU	Mean (SD)	na	35.9 (11.0)	25.3 (12.4)	23.1 (17.9)	19.2 (12.2)	0.161
		Median (Q1 – Q3)	na	35.9 (28.1 – 43.8)	28.1 (0.0 – 43.8)	18.8 (0.0 – 78.1)	17.2 (0.0 – 46.9)	
	Anxiety - Blood test	Mean (SD)	na	0.0 (0.0)	13.2 (21.4)	14.7 (17.2)	9.7 (13.6)	0.266
		Median (Q1 – Q3)	na	0.0 (0.0 – 0.0)	0.0 (0.0 – 50.0)	12.5 (0.0 – 62.5)	0.0 (0.0 – 50.0)	
	Anxiety - Phe levels	Mean (SD)	na	50.0 (35.4)	40.3 (31.1)	48.8 (38.4)	44.4 (39.4)	0.902
		Median (Q1 – Q3)	na	50.0 (25.0 – 75.0)	25.0 (0.0 – 100.0)	50.0 (0.0 – 100.0)	50.0 (0.0 – 100.0)	

Table 2_a : Comparison of the Child PKU-QOL scores according to overall assessment of health status (cont‘d)

Modules	Domains		Poor (N=0)	Fair (N=2)	Good (N=19)	Very good (N=41)	Excellent (N=28)	p-value*
**Supplement administration**	Adherence to supplements	**Mean (SD)**	na	50.0 (70.7)	8.8 (13.1)	10.9 (15.2)	9.9 (14.3)	0.871
		**Median (Q1 – Q3)**	na	50.0 (0.0 – 100.0)	0.0 (0.0 – 37.5)	0.0 (0.0 – 50.0)	0.0 (0.0 – 50.0)	
	Practical impact of supplements	**Mean (SD)**	na	50.0 (70.7)	26.5 (40.0)	17.3 (29.3)	13.0 (25.1)	0.571
		**Median (Q1 – Q3)**	na	50.0 (0.0 – 100.0)	0.0 (0.0 – 100.0)	0.0 (0.0 – 100.0)	0.0 (0.0 – 75.0)	
	Guilt if poor adherence to supplements	**Mean (SD)**	na	12.5 (17.7)	41.2 (35.3)	51.3 (39.0)	41.3 (40.3)	0.444
		**Median (Q1 – Q3)**	na	12.5 (0.0 – 25.0)	25.0 (0.0 – 100.0)	50.0 (0.0 – 100.0)	25.0 (0.0 – 100.0)	
	Relationships within family because of supplements	**Mean (SD)**	na	25.0 (35.4)	7.4 (14.7)	13.5 (27.4)	4.0 (11.8)	0.364
		**Median (Q1 – Q3)**	na	25.0 (0.0 – 50.0)	0.0 (0.0 – 50.0)	0.0 (0.0 – 100.0)	0.0 (0.0 – 50.0)	
	Taste - Supplements	**Mean (SD)**	na	0.0 (0.0)	29.4 (29.6)	32.7 (29.9)	47.8 (32.8)	0.063
		**Median (Q1 – Q3)**	na	0.0 (0.0 – 0.0)	25.0 (0.0 – 100.0)	25.0 (0.0 – 100.0)	50.0 (0.0 – 100.0)	
**Dietary protein restriction**	Food temptations	**Mean (SD)**	na	31.3 (8.8)	23.4 (29.2)	30.1 (27.8)	34.1 (28.2)	0.545
		**Median (Q1 – Q3)**	na	31.3 (25.0 – 37.5)	18.8 (0.0 – 100.0)	25.0 (0.0 – 100.0)	31.3 (0.0 – 87.5)	
	Adherence to dietary protein restriction	**Mean (SD)**	na	37.5 (17.7)	9.4 (13.3)	11.2 (16.4)	7.4 (16.7)	0.059
		**Median (Q1 – Q3)**	na	37.5 (25.0 – 50.0)	0.0 (0.0 – 37.5)	0.0 (0. – 62.5)	0.0 (0.0 – 62.5)	
	Social impact of dietary protein restriction	**Mean (SD)**	na	22.5 (24.7)	21.1 (19.3)	19.2 (19.6)	17.0 (17.8)	0.884
		**Median (Q1 – Q3)**	na	22.5 (5.0 – 40.0)	17.5 (0.0 – 62.5)	15.0 (0.0 – 80.0)	15.0 (0.0 – 60.0)	
	Taste – low protein food	**Mean (SD)**	na	0.0 (0.0)	17.2 (19.8)	20.3 (21.9)	18.2 (29.1)	0.390
		**Median (Q1 – Q3)**	na	0.0 (0.0 – 0.0)	12.5 (0.0 – 50.0)	25.0 (0.0 – 100.0)	0.0 (0.0 – 100.0)	
	Food enjoyment	**Mean (SD)**	na	50.0 (70.7)	11.1 (15.4)	20.6 (22.6)	13.9 (24.4)	0.268
		**Median (Q1 – Q3)**	na	50.0 (0.0 – 100.0)	0.0 (0.0 – 50.0)	25.0 (0.0 – 75.0)	0.0 (0.0 – 100.0)	
	Guilt if dietary protein restriction not followed	**Mean (SD)**	na	62.5 (53.0)	57.8 (35.0)	53.9 (41.3)	49.0 (44.8)	0.870
		**Median (Q1 – Q3)**	na	62.5 (25.0 – 100.0)	62.5 (0.0 – 100.0)	50.0 (0.0 – 100.0)	50.0 (0.0 – 100.0)	
	Overall difficulty following dietary protein restriction	**Mean (SD)**	na	0.0 (0.0)	18.8 (23.3)	21.2 (27.2)	17.6 (32.4)	0.362
		**Median (Q1 – Q3)**	na	0.0 (0.0 – 0.0)	12.5 (0.0 – 75.0)	25.0 (0.0 – 100.0)	0.0 (0.0 – 100.0)	

**Non-parametric P-value for between-group comparisons: Kruskal-Wallis*
In bold p-value<0.05

Table 2_b : Comparison of the Adolescent PKU-QOL scores according to overall assessment of health status

Modules	Domains		Poor (N=0)	Fair (N=0)	Good (N=38)	Very good (N=42)	Excellent (N=30)	p-value*
**Symptoms**	Self-rated status health	**Mean (SD)**	na	na	39.3 (21.3)	41.3 (23.0)	20.8 (24.6)	**0.001**
		**Median (Q1 – Q3)**	na	na	50.0 (0.0 – 75.0)	50.0 (0.0 – 75.0)	12.5 (0.0 – 75.0)	
	Headaches	**Mean (SD)**	na	na	18.6 (24.5)	20.6 (25.2)	20.8 (27.1)	0.908
		**Median (Q1 – Q3)**	na	na	0.0 (0.0 – 75.0)	12.5 (0.0 – 100.0)	12.5 (0.0 – 100.0)	
	Stomach aches	**Mean (SD)**	na	na	16.4 (21.8)	15.6 (20.2)	10.8 (21.5)	0.355
		**Median (Q1 – Q3)**	na	na	0.0 (0.0 – 75.0)	0.0 (0.0 – 75.0)	0.0 (0.0 – 100.0)	
	Tiredness	**Mean (SD)**	na	na	43.6 (28.0)	41.9 (23.6)	35.0 (26.7)	0.264
		**Median (Q1 – Q3)**	na	na	50.0 (0.0 – 100.0)	50.0 (0.0 – 100.0)	25.0 (0.0 – 100.0)	
	Irritability	**Mean (SD)**	na	na	32.9 (26.3)	20.2 (25.4)	21.7 (24.3)	0.062
		**Median (Q1 – Q3)**	na	na	50.0 (0.0 – 75.0)	0.0 (0.0 – 75.0)	25.0 (0.0 – 100.0)	
	Aggressiveness	**Mean (SD)**	na	na	17.1 (26.3)	11.3 (24.2)	6.7 (13.0)	0.305
		**Median (Q1 – Q3)**	na	na	0.0 (0.0 – 75.0)	0.0 (0.0 – 100.0)	0.0 (0.0 – 50.0)	
	Moodiness	**Mean (SD)**	na	na	24.3 (24.6)	16.7 (21.9)	19.2 (21.5)	0.337
		**Median (Q1 – Q3)**	na	na	25.0 (0.0 – 75.0)	0.0 (0.0 – 75.0)	12.5 (0.0 – 50.0)	
	Sadness	**Mean (SD)**	na	na	14.3 (20.4)	11.9 (20.1)	10.0 (18.1)	0.570
		**Median (Q1 – Q3)**	na	na	0.0 (0.0 – 75.0)	0.0 (0.0 – 50.0)	0.0 (0.0 – 50.0)	
	Anxiety	**Mean (SD)**	na	na	20.0 (25.6)	10.7 (20.0)	11.7 (25.2)	0.077
		**Median (Q1 – Q3)**	na	na	0.0 (0.0 – 100.0)	0.0 (0.0 – 75.0)	0.0 (0.0 – 100.0)	
	Lack of concentration	**Mean (SD)**	na	na	25.7 (22.3)	16.7 (23.9)	16.7 (26.5)	0.057
		**Median (Q1 – Q3)**	na	na	25.0 (0.0 – 75.0)	0.0 (0.0 – 75.0)	0.0 (0.0 – 100.0)	
	Slow thinking	**Mean (SD)**	na	na	15.7 (19.3)	11.9 (20.1)	14.2 (20.4)	0.549
		**Median (Q1 – Q3)**	na	na	0.0 (0.0 – 50.0)	0.0 (0.0 – 75.0)	0.0 (0.0 – 50.0)	
**PKU in general**	Emotional impact of PKU	**Mean (SD)**	na	na	38.9 (23.0)	28.2 (17.6)	29.7 (17.2)	0.165
		**Median (Q1 – Q3)**	na	na	30.0 (0.0 – 90.0)	30.0 (0.0 – 70.0)	30.0 (0.0 – 70.0)	
	Practical impact of PKU	**Mean (SD)**	na	na	19.3 (17.1)	9.0 (11.0)	8.3 (11.2)	0.095
		**Median (Q1 – Q3)**	na	na	16.7 (0.0 – 50.0)	8.3 (0.0 – 33.3)	8.3 (0.0 – 33.3)	
	Social impact of PKU	**Mean (SD)**	na	na	18.1 (18.1)	14.8 (12.7)	13.8 (11.6)	0.723
		**Median (Q1 – Q3)**	na	na	16.7 (0.0 – 91.7)	8.3 (0.0 – 50.0)	8.3 (0.0 – 33.3)	
	Overall impact of PKU	**Mean (SD)**	na	na	28.4 (18.8)	19.9 (12.1)	20.2 (12.7)	0.093
		**Median (Q1 – Q3)**	na	na	25.0 (0.0 – 85.0)	20.0 (2.3 – 50.0)	17.5 (0.0 – 55.0)	
	Anxiety - Blood test	**Mean (SD)**	na	na	13.6 (25.3)	9.8 (19.5)	9.2 (16.1)	0.990
		**Median (Q1 – Q3)**	na	na	0.0 (0.0 – 100.0)	0.0 (0.0 – 87.5)	0.0 (0.0 – 50.0)	
	Anxiety - Phe levels	**Mean (SD)**	na	na	40.7 (33.3)	33.5 (31.9)	32.5 (29.5)	0.530
		**Median (Q1 – Q3)**	na	na	50.0 (0.0 – 100.0)	25.0 (0.0 – 100.0)	25.0 (0.0 – 100.0)	

Table 2_b : Comparison of the Adolescent PKU-QOL scores according to overall assessment of health status (cont'd)

Modules	Domains		Poor (N=0)	Fair (N=0)	Good (N=38)	Very good (N=42)	Excellent (N=30)	p-value*
**Supplement administration**	Adherence to supplements	**Mean (SD)**	na	na	21.4 (25.1)	11.1 (12.9)	11.5 (16.4)	0.239
		**Median (Q1 – Q3)**	na	na	8.3 (0.0 – 83.3)	6.3 (0.0 – 50.0)	6.3 (0.0 -66.7)	
	Practical impact of supplements	**Mean (SD)**	na	na	24.0 (28.6)	14.2 (16.9)	20.3 (24.4)	0.447
		**Median (Q1 – Q3)**	na	na	12.5 (0.0 – 100.0)	12.5 (0.0 – 62.5)	6.3 (0.0 – 75.0)	
	Guilt if poor adherence to supplements	**Mean (SD)**	na	na	40.3 (34.0)	43.9 (37.5)	46.2 (36.5)	0.859
		**Median (Q1 – Q3)**	na	na	25.0 (0.0 – 100.0)	25.0 (0.0 – 100.0)	50.0 (0.0 – 100.0)	
	Relationships within family because of supplements	**Mean (SD)**	na	na	14.8 (28.3)	14.9 (26.0)	13.0 (20.6)	0.983
		**Median (Q1 – Q3)**	na	na	0.0 (0.0 – 100.0)	0.0 (0.0 – 100.0)	0.0 (0.0 – 50.0)	
	Taste - Supplements	**Mean (SD)**	na	na	44.5 (26.0)	39.9 (23.2)	38.0 (26.1)	0.634
		**Median (Q1 – Q3)**	na	na	50.0 (0.0 – 100.0)	50.0 (0.0 – 100.0)	50.0 (0.0 – 100.0)	
**Dietary protein restriction**	Food temptations	**Mean (SD)**	na	na	30.8 (31.6)	27.0 (26.4)	19.6 (25.5)	0.360
		**Median (Q1 – Q3)**	na	na	25.0 (0.0 – 100.0)	25.0 (0.0 – 100.0)	12.5 (0.0 – 87.5)	
	Adherence to dietary protein restriction	**Mean (SD)**	na	na	21.3 (25.6)	10.7 (12.4)	7.6 (12.6)	0.083
		**Median (Q1 – Q3)**	na	na	12.5 (0.0 – 83.3)	8.3 (0.0 – 50.0)	0.0 (0.0 – 50.0)	
	Practical impact of dietary protein restriction	**Mean (SD)**	na	na	30.7 (21.8)	24.6 (16.4)	25.9 (14.7)	0.659
		**Median (Q1 – Q3)**	na	na	26.8 (0.0 – 85.7)	24.3 (0.0 – 64.3)	28.6 (0.0 – 67.9)	
	Social impact of dietary protein restriction	**Mean (SD)**	na	na	19.4 (25.2)	11.1 (18.3)	10.7 (15.2)	0.356
		**Median (Q1 – Q3)**	na	na	7.5 (0.0 – 90.0)	5.0 (0.0 – 80.0)	5.0 (0.0 – 65.0)	
	Overall impact of dietary protein restriction	**Mean (SD)**	na	na	26.0 (21.1)	17.2 (15.1)	18.3 (12.8)	0.177
		**Median (Q1 – Q3)**	na	na	19.4 (0.0 – 86.4)	13.1 (0.0 – 59.1)	18.2 (0.0 – 52.3)	
	Taste – low protein food	**Mean (SD)**	na	na	33.1 (31.2)	19.1 (17.5)	26.3 (25.6)	0.228
		**Median (Q1 – Q3)**	na	na	25.0 (0.0 – 100.0)	25.0 (0.0 – 50.0)	25.0 (0.0 – 100.0)	
	Food enjoyment	**Mean (SD)**	na	na	15.8 (26.7)	14.2 (26.7)	19.6 (27.1)	0.461
		**Median (Q1 – Q3)**	na	na	0.0 (0.0 – 100.0)	0.0 (0.0 – 100.0)	0.0 (0.0 – 100.0)	
	Guilt if dietary protein restriction not followed	**Mean (SD)**	na	na	44.4 (35.8)	50.6 (38.3)	42.4 (30.6)	0.657
		**Median (Q1 – Q3)**	na	na	50.0 (0.0 – 100.0)	50.0 (0.0 – 100.0)	50.0 (0.0 – 100.0)	
	Overall difficulty following dietary protein restriction	**Mean (SD)**	na	na	21.2 (30.1)	15.8 (23.6)	13.0 (26.0)	0.458
		**Median (Q1 – Q3)**	na	na	0.0 (0.0 – 100.0)	0.0 (0.0 – 100.0)	0.0 (0.0 – 100.0)	

**Non-parametric P-value for between-group comparisons: Kruskal-Wallis*
In bold p-value<0.05

Table 2_c : Comparison of the Adult PKU-QOL scores according to overall assessment of health status

Modules	Domains		Poor (N=0)	Fair (N=3)	Good (N=28)	Very good (N=46)	Excellent (N=26)	p-value*
**Symptoms**	Self-rated health status	**Mean (SD)**	na	66.7 (28.9)	46.3 (16.6)	30.4 (21.0)	22.1 (22.7)	**<0.001**
		**Median (Q1 – Q3)**	na	50.0 (50.0 – 100.0)	50.0 (25.0 – 75.0)	25.0 (0.0 – 75.0)	25.0 (0.0 – 50.0)	
	Headaches	**Mean (SD)**	na	33.3 (28.9)	26.9 (25.4)	19.0 (24.3)	18.3 (20.7)	0.395
		**Median (Q1 – Q3)**	na	50.0 (0.0 – 50.0)	25.0 (0.0 – 75.0)	0.0 (0.0 – 75.0)	12.5 (0.0 – 50.0)	
	Stomach aches	**Mean (SD)**	na	50.0 (25.0)	26.0 (32.0)	13.6 (22.8)	9.6 (18.8)	**0.013**
		**Median (Q1 – Q3)**	na	50.0 (25.0 – 75.0)	12.5 (0.0 – 100.0)	0.0 (0.0 – 75.0)	0.0 (0.0 – 50.0)	
	Tiredness	**Mean (SD)**	na	41.7 (14.4)	51.0 (26.9)	44.0 (27.5)	38.5 (29.4)	0.479
		**Median (Q1 – Q3)**	na	50.0 (25.0 – 50.0)	50.0 (0.0 – 100.0)	50.0 (0.0 – 100.0)	37.5 (0.0 – 100.0)	
	Trembling hands	**Mean (SD)**	na	16.7 (28.9)	15.4 (22.4)	10.3 (22.7)	13.5 (24.7)	0.517
		**Median (Q1 – Q3)**	na	0.0 (0.0 – 50.0)	0.0 (0.0 – 75.0)	0.0 (0.0 – 75.0)	0.0 (0.0 – 100.0)	
	Irritability	**Mean (SD)**	na	66.7 (14.4)	35.2 (25.2)	34.2 (22.6)	26.9 (27.3)	**0.050**
		**Median (Q1 – Q3)**	na	75.0 (50.0 – 75.0)	50.0 (0.0 – 75.0)	50.0 (0.0 – 75.0)	25.0 (0.0 – 100.0)	
	Aggressiveness	**Mean (SD)**	na	41.7 (38.2)	14.4 (21.4)	9.2 (17.8)	13.5 (23.7)	0.243
		**Median (Q1 – Q3)**	na	50.0 (0.0 – 75.0)	0.0 (0.0 – 50.0)	0.0 (0.0 – 75.0)	0.0 (0.0 – 75.0)	
	Moodiness	**Mean (SD)**	na	33.3 (28.9)	27.9 (24.8)	23.4 (25.5)	16.3 (23.4)	0.297
		**Median (Q1 – Q3)**	na	50.0 (0.0 – 50.0)	25.0 (0.0 – 75.0)	25.0 (0.0 – 75.0)	0.0 (0.0 – 75.0)	
	Sadness	**Mean (SD)**	na	50.0 (25.0)	40.4 (30.9)	26.1 (26.3)	21.2 (26.2)	**0.044**
		**Median (Q1 – Q3)**	na	50.0 (25.0 – 75.0)	50.0 (0.0 – 100.0)	25.0 (0.0 – 100.0)	0.0 (0.0 – 75.0)	
	Anxiety	**Mean (SD)**	na	50.0 (43.3)	24.0 (28.7)	15.2 (23.9)	29.8 (34.7)	0.130
		**Median (Q1 – Q3)**	na	75.0 (0.0 – 75.0)	12.5 (0.0 – 75.0)	0.0 (0.0 – 75.0)	25.0 (0.0 – 100.0)	
	Lack of concentration	**Mean (SD)**	na	33.3 (14.4)	31.7 (27.9)	23.4 (25.5)	17.3 (25.3)	0.160
		**Median (Q1 – Q3)**	na	25.0 (25.0 – 50.0)	25.0 (0.0 – 100.0)	25.0 (0.0 – 100.0)	0.0 (0.0 – 75.0)	
	Slow thinking	**Mean (SD)**	na	25.0 (25.0)	28.8 (30.6)	15.2 (25.0)	11.5 (23.7)	**0.038**
		**Median (Q1 – Q3)**	na	25.0 (0.0 – 50.0)	25.0 (0.0 – 100.0)	0.0 (0.0 – 100.0)	0.0 (0.0 – 75.0)	

Table 2_c : Comparison of the Adult PKU-QOL scores according to overall assessment of health status (cont'd)

Modules	Domains		Poor (N=0)	Fair (N=3)	Good (N=28)	Very good (N=46)	Excellent (N=26)	p-value*
PKU in general	Emotional impact of PKU	Mean (SD)	na	55.0 (10.0)	58.5 (16.7)	43.0 (22.0)	32.5 (20.6)	**<0.001**
		Median (Q1 – Q3)	na	55.0 (45.0 – 65.0)	60.0 (20.0 – 85.0)	45.0 (0.0 – 100.0)	30.0 (0.0 – 95.0)	
	Practical impact of PKU	Mean (SD)	na	21.5 (14.8)	21.9 (17.8)	20.0 (17.6)	9.9 (13.0)	**0.023**
		Median (Q1 – Q3)	na	18.8 (8.3 – 37.5)	25.0 (0.0 – 58.3)	16.7 (0.0 – 75.0)	6.3 (0.0 – 43.8)	
	Social impact of PKU	Mean (SD)	na	27.8 (30.1)	23.0 (19.2)	17.1 (13.5)	12.3 (10.7)	0.173
		Median (Q1 – Q3)	na	12.5 (8.3 – 62.5)	18.8 (0.0 – 68.8)	14.6 (0.0 – 43.8)	10.4 (0.0 – 41.7)	
	Overall impact of PKU	Mean (SD)	na	41.7 (14.7)	39.1 (14.2)	28.8 (15.5)	21.3 (12.5)	**0.004**
		Median (Q1 – Q3)	na	41.7 (31.3 – 52.1)	39.6 (12.5 – 68.8)	27.1 (2.1 – 62.5)	17.7 (6.3 – 52.1)	
	Anxiety - Blood test	Mean (SD)	na	18.8 (8.8)	6.0 (13.5)	14.2 (27.8)	8.3 (17.2)	0.218
		Median (Q1 – Q3)	na	18.8 (12.5 – 25.0)	0.0 (0.0 – 50.0)	0.0 (0.0 – 100.0)	0.0 (0.0 – 75.0)	
	Anxiety - Phe levels	Mean (SD)	na	75.0 (25.0)	35.2 (28.8)	34.8 (25.0)	37.5 (28.5)	0.170
		Median (Q1 – Q3)	na	75.0 (50.0 – 100.0)	25.0 (0.0 – 100.0)	25.0 (0.0 – 100.0)	25.0 (0.0 – 75.0)	
	Anxiety - Phe levels during pregnancy	Mean (SD)	na	33.3 (38.2)	86.5 (16.5)	83.6 (22.5)	79.7 (24.5)	0.092
		Median (Q1 – Q3)	na	25.0 (0.0 – 75.0)	100.0 (50.0 – 100.0)	100.0 (25.0 – 100.0)	100.0 (50.0 – 100.0)	
	Financial impact of PKU	Mean (SD)	na	41.7 (52.0)	30.6 (31.3)	14.1 (23.4)	13.5 (22.6)	**0.035**
		Median (Q1 – Q3)	na	25.0 (0.0 – 100.0)	25.0 (0.0 – 100.0)	0.0 (0.0 – 100.0)	0.0 (0.0 – 75.0)	
	Information on PKU	Mean (SD)	na	50.0 (50.0)	38.9 (23.3)	34.2 (25.5)	27.9 (19.1)	0.265
		Median (Q1 – Q3)	na	50.0 (0.0 – 100.0)	50.0 (0.0 – 100.0)	25.0 (0.0 – 100.0)	25.0 (0.0 – 75.0)	
Supplement administration	Adherence to supplements	Mean (SD)	na	16.7 (11.8)	20.2 (20.1)	19.3 (18.1)	21.8 (25.1)	0.991
		Median (Q1 – Q3)	na	16.7 (8.3 – 25.0)	16.7 (0.0 – 66.7)	16.7 (0.0 – 66.7)	8.3 (0.0 – 66.47)	
	Practical impact of supplements	Mean (SD)	na	60.4 (20.1)	28.5 (26.8)	18.8 (17.7)	15.9 (14.7)	**0.033**
		Median (Q1 – Q3)	na	68.8 (37.5 – 75.0)	18.8 (0.0 – 100.0)	18.8 (0.0 – 56.3)	12.5 (0.0 – 37.5)	
	Guilt if poor adherence to supplements	Mean (SD)	na	41.7 (52.0)	46.7 (34.0)	49.3 (35.1)	47.2 (27.0)	0.969
		Median (Q1 – Q3)	na	25.0 (0.0 – 100.0)	50.0 (0.0 – 100.0)	50.0 (0.0 – 100.0)	50.0 (0.0 – 75.0)	
	Relationships within family because of supplements	Mean (SD)	na	8.3 (14.4)	9.1 (26.2)	12.8 (22.5)	10.0 (18.8)	0.634
		Median (Q1 – Q3)	na	0.0 (0.0 – 25.0)	0.0 (0.0 – 100.0)	0.0 (0.0 – 75.0)	0.0 (0.0 – 75.0)	
	Taste - Supplements	Mean (SD)	na	58.3 (14.4)	45.2 (33.2)	48.6 (19.5)	55.3 (28.4)	0.543
		Median (Q1 – Q3)	na	50.0 (50.0 – 75.0)	50.0 (0.0 – 100.0)	50.0 (25.0 – 100.0)	50.0 (0.0 – 100.0)	

Table 2_c : Comparison of the Adult PKU-QOL scores according to overall assessment of health status (cont'd)

Modules	Domains		Poor (N=0)	Fair (N=3)	Good (N=28)	Very good (N=46)	Excellent (N=26)	p-value*
**Dietary protein restriction**	Food temptations	**Mean (SD)**	na	50.0 (12.5)	45.3 (29.2)	34.5 (22.4)	23.7 (19.0)	**0.038**
		**Median (Q1 – Q3)**	na	50.0 (37.5 – 62.5)	50.0 (0.0 – 100.0)	43.8 (0.0 – 75.0)	25.0 (0.0 – 50.0)	
	Adherence to dietary protein restriction	**Mean (SD)**	na	12.5 (17.7)	21.2 (17.4)	21.0 (18.4)	16.4 (19.7)	0.671
		**Median (Q1 – Q3)**	na	12.5 (0.0 – 25.0)	18.8 (0.0 – 56.3)	22.5 (0.0 – 55.0)	10.0 (0.0 56.3)	
	Social impact of dietary protein restriction	**Mean (SD)**	na	51.4 (31.8)	21.7 (19.8)	15.4 (15.1)	13.9 (16.7)	0.064
		**Median (Q1 – Q3)**	na	58.3 (16.7 – 79.2)	16.7 (0.0 – 75.0)	11.3 (0.0 – 54.2)	8.3 (0.0 – 54.2)	
	Practical impact of dietary protein restriction	**Mean (SD)**	na	64.3 (15.6)	38.5 (16.5)	31.7 (18.6)	33.7 (23.0)	0.079
		**Median (Q1 – Q3)**	na	71.4 (46.4 – 75.0)	39.6 (15.0 – 71.4)	30.4 (0.0 – 64.3)	32.1 (7.1 – 67.9)	
	Overall impact of dietary protein restriction	**Mean (SD)**	na	58.3 (22.5)	29.5 (14.9)	23.9 (15.0)	23.7 (18.8)	0.063
		**Median (Q1 – Q3)**	na	67.3 (32.7 – 75.0)	27.1 (9.1 – 58.3)	22.8 (0.0 – 53.8)	20.8 (2.5 – 59.6)	
	Taste – low protein food	**Mean (SD)**	na	58.3 (14.4)	36.8 (25.5)	30.5 (19.8)	30.4 (17.5)	0.125
		**Median (Q1 – Q3)**	na	50.0 (50.0 – 75.0)	25.0 (0.0 – 100.0)	25.0 (0.0 – 75.0)	25.0 (0.0 – 50.0)	
	Food enjoyment	**Mean (SD)**	na	58.3 (52.0)	29.3 (28.9)	25.7 (29.3)	18.4 (26.1)	0.335
		**Median (Q1 – Q3)**	na	75.0 (0.0 – 100.0)	25.0 (0.0 – 100.0)	25.0 (0.0 – 100.0)	0.0 (0.0 – 100.0)	
	Guilt if dietary protein restriction not followed	**Mean (SD)**	na	50.0 (43.3)	50.0 (29.9)	57.2 (35.3)	52.5 (26.8)	0.854
		**Median (Q1 – Q3)**	na	25.0 (25.0 – 100.0)	50.0 (0.0 – 100.0)	50.0 (0.0 – 100.0)	50.0 (0.0 – 100.0)	
	Overall difficulty following dietary protein restriction	**Mean (SD)**	na	41.7 (38.2)	34.8 (29.9)	23.1 (25.9)	28.9 (29.2)	0.383
		**Median (Q1 – Q3)**	na	50.0 (0.0 – 75.0)	25.0 (0.0 – 100.0)	25.0 (0.0 – 100.0)	25.0 (0.0 – 75.0)	

**Non-parametric P-value for between-group comparisons: Kruskal-Wallis*
In bold p-value<0.05

Table 2_d : Comparison of the Parent PKU-QOL scores according to patient's overall assessment of health status

Modules	Domains		Poor (N=0)	Fair (N=2)	Good (N=64)	Very good (N=101)	Excellent (N=84)	p-value*
Symptoms	Child health status	**Mean (SD)**	na	12.5 (17.7)	38.5 (19.7)	34.3 (25.4)	23.2 (22.9)	**<0.001**
		**Median (Q1 – Q3)**		12.5 (0.0 – 25.0)	50.0 (0.0 – 75.0)	25.0 (0.0 – 100.0)	25.0 (0.0 – 100.0)	
	Headaches	**Mean (SD)**	na	25.0 (35.4)	20.1 (23.2)	15.4 (20.7)	9.2 (17.7)	**0.012**
		**Median (Q1 – Q3)**		25.0 (0.0 – 50.0)	25.0 (0.0 – 75.0)	0.0 (0.0 – 75.0)	0.0 (0.0 – 75.0)	
	Stomach aches	**Mean (SD)**	na	37.5 (53.0)	17.6 (22.5)	21.0 (24.1)	16.7 (25.2)	0.391
		**Median (Q1 – Q3)**		37.5 (0.0 – 75.0)	0.0 (0.0 – 75.0)	25.0 (0.0 – 75.0)	0.0 (0.0 – 100.0)	
	Tiredness	**Mean (SD)**	na	12.5 (17.7)	35.7 (25.2)	31.3 (23.8)	30.1 (26.3)	0.313
		**Median (Q1 – Q3)**		12.5 (0.0 – 25.0)	50.0 (0.0 – 100.0)	25.0 (0.0 – 100.0)	25.0 (0.0 – 100.0)	
	Irritability	**Mean (SD)**	na	62.5 (17.7)	43.9 (27.3)	34.8 (26.7)	31.3 (27.7)	**0.015**
		**Median (Q1 – Q3)**		62.5 (50.0 – 75.0)	50.0 (0.0 – 100.0)	25.0 (0.0 – 100.0)	25.0 (0.0 – 100.0)	
	Aggressiveness	**Mean (SD)**	na	37.5 (17.7)	16.9 (24.3)	11.5 (21.0)	11.6 (20.9)	0.085
		**Median (Q1 – Q3)**		37.5 (25.0 – 50.0)	0.0 (0.0 – 100.0)	0.0 (0.0 – 75.0)	0.0 (0.0 – 100.0)	
	Moodiness	**Mean (SD)**	na	12.5 (17.7)	32.0 (24.2)	25.8 (26.2)	27.7 (26.2)	0.291
		**Median (Q1 – Q3)**		12.5 (0.0 – 25.0)	25.0 (0.0 – 100.0)	25.0 (0.0 – 100.0)	25.0 (0.0 – 100.0)	
	Sadness	**Mean (SD)**	na	25.0 (0.0)	25.4 (21.6)	18.9 (22.6)	12.2 (17.7)	**0.001**
		**Median (Q1 – Q3)**		25.0 (25.0 – 25.0)	25.0 (0.0 – 100.0)	25.0 (0.0 – 100.0)	0.0 (0.0 – 75.0)	
	Anxiety	**Mean (SD)**	na	0.0 (0.0)	20.8 (26.7)	13.4 (20.7)	10.8 (18.4)	0.066
		**Median (Q1 – Q3)**		0.0 (0.0 – 0.0)	0.0 (0.0 – 100.0)	0.0 (0.0 – 100.0)	0.0 (0.0 – 75.0)	
	Lack of concentration	**Mean (SD)**	na	12.5 (17.7)	36.7 (30.7)	28.4 (27.2)	28.4 (27.1)	0.278
		**Median (Q1 – Q3)**		12.5 (0.0 – 25.0)	25.0 (0.0 – 100.0)	25.0 (0.0 – 100.0)	25.0 (0.0 – 100.0)	
	Slow thinking	**Mean (SD)**	na	12.5 (17.7)	27.6 (28.4)	18.0 (26.3)	16.3 (23.9)	0.053
		**Median (Q1 – Q3)**		12.5 (0.0 – 25.0)	25.0 (0.0 – 100.0)	0.0 (0.0 – 100.0)	0.0 (0.0 – 75.0)	

Table 2_d : Comparison of the Parent PKU-QOL scores according to patient's overall assessment of health status (cont'd)

Modules	Domains		Poor (N=0)	Fair (N=2)	Good (N=64)	Very good (N=101)	Excellent (N=84)	p-value*
**PKU in general**	Emotional impact of PKU	**Mean (SD)**	na	28.1 (13.3)	52.4 (22.4)	41.6 (21.8)	37.3 (22.9)	**<0.001**
		**Median (Q1 – Q3)**		28.1 (18.8 – 37.5)	56.3 (6.3 – 87.5)	37.5 (6.3 – 100.0)	34.4 (0.0 – 100.0)	
	Practical impact of PKU	**Mean (SD)**	na	25.4 (28.9)	18.9 (15.8)	14.9 (13.8)	10.4 (13.0)	**0.007**
		**Median (Q1 – Q3)**		25.4 (5.0 – 45.8)	16.7 (0.0 – 70.0)	12.5 (0.0 – 50.0)	5.0 (0.0 – 54.2)	
	Social impact of PKU	**Mean (SD)**	na	25.0 (21.2)	21.7 (17.5)	16.6 (17.0)	14.4 (15.3)	**0.019**
		**Median (Q1 – Q3)**		25.0 (10.0 – 40.0)	15.0 (0.0 – 90.0)	10.0 (0.0 – 80.0)	10.0 (0.0 – 70.0)	
	Overall impact of PKU	**Mean (SD)**	na	26.2 (21.9)	30.6 (15.9)	23.5 (14.9)	19.6 (14.2)	**<0.001**
		**Median (Q1 – Q3)**		26.2 (10.7 – 41.7)	28.3 (6.7 – 76.9)	21.2 (1.7 – 67.3)	15.8 (0.0 – 73.1)	
	Child anxiety - Blood test	**Mean (SD)**	na	25.0 (0.0)	26.0 (27.9)	22.3 (28.5)	23.8 (27.5)	0.670
		**Median (Q1 – Q3)**		25.0 (25.0 – 25.0)	12.5 (0.0 – 100.0)	12.5 (0.0 – 100.0)	12.5 (0.0 – 100.0)	
	Impact of child anxiety - Blood test	**Mean (SD)**	na	25.0 (17.7)	28.4 (31.2)	23.3 (27.2)	23.8 (28.7)	0.784
		**Median (Q1 – Q3)**		25.0 (12.5 – 37.5)	25.0 (0.0 – 100.0)	12.5 (0.0 – 100.0)	12.5 (0.0 – 100.0)	
	Anxiety - Phe levels	**Mean (SD)**	na	50.0 (35.4)	61.9 (28.7)	54.6 (30.7)	50.6 (34.9)	0.206
		**Median (Q1 – Q3)**		50.0 (25.0 – 75.0)	75.0 (0.0 – 100.0)	50.0 (0.0 – 100.0)	50.0 (0.0 – 100.0)	
	Financial impact of PKU	**Mean (SD)**	na	75.0 (35.4)	30.0 (30.5)	30.2 (30.0)	18.2 (24.3)	**0.004**
		**Median (Q1 – Q3)**		75.0 (50.0 – 100.0)	25.0 (0.0 – 100.0)	25.0 (0.0 – 100.0)	0.0 (0.0 – 100.0)	
	Information on PKU	**Mean (SD)**	na	37.5 (53.0)	38.1 (25.7)	31.1 (23.3)	26.5 (23.4)	**0.032**
		**Median (Q1 – Q3)**		37.5 (0.0 – 75.0)	25.0 (0.0 – 100.0)	25.0 (0.0 – 100.0)	25.0 (0.0 – 100.0)	
**Supplement administration**	Adherence to supplements	**Mean (SD)**	na	0.0 (0.0)	16.8 (24.1)	9.9 (14.9)	10.0 (16.2)	0.224
		**Median (Q1 – Q3)**		0.0 (0.0 – 0.0)	0.0 (0.0 – 100.0)	0.0 (0.0 – 50.0)	0.0 (0.0 – 75.0)	
	Management of supplements	**Mean (SD)**	na	37.5 (17.7)	15.5 (27.8)	20.8 (29.5)	14.8 (24.5)	0.180
		**Median (Q1 – Q3)**		37.5 (25.0 – 50.0)	0.0 (0.0 – 100.0)	0.0 (0.0 – 100.0)	0.0 (0.0 – 100.0)	
	Practical impact of supplements	**Mean (SD)**	na	25.0 (11.8)	21.5 (26.3)	22.0 (22.3)	18.1 (23.2)	0.353
		**Median (Q1 – Q3)**		25.0 (16.7 – 33.3)	8.3 (0.0 – 100.0)	16.7 (0.0 – 100.0)	8.3 (0.0 – 83.3)	
	Guilt if poor adherence to supplements	**Mean (SD)**	na	62.5 (53.0)	55.8 (36.3)	53.4 (33.8)	49.3 (32.9)	0.697
		**Median (Q1 – Q3)**		62.5 (25.0 – 100.0)	50.0 (0.0 – 100.0)	50.0 (0.0 – 100.0)	50.0 (0.0 – 100.0)	
	Relationships within family because of supplements	**Mean (SD)**	na	12.5 (17.7)	19.2 (27.4)	23.3 (28.4)	14.3 (21.7)	0.246
		**Median (Q1 – Q3)**		12.5 (0.0 – 25.0)	0.0 (0.0 – 100.0)	25.0 (0.0 – 100.0)	0.0 (0.0 – 75.0)	

Table 2_d : Comparison of the Parent PKU-QOL scores according to patient's overall assessment of health status (cont'd)

Modules	Domains		Poor (N=0)	Fair (N=2)	Good (N=64)	Very good (N=101)	Excellent (N=84)	p-value*
**Dietary protein restriction**	Adherence to dietary protein restriction	**Mean (SD)**	na	12.5 (17.7)	20.5 (26.8)	15.9 (28.5)	5.7 (16.0)	**0.001**
		**Median (Q1 – Q3)**	na	12.5 (0.0 – 25.0)	0.0 (0.0 – 100.0)	0.0 (0.0 – 100.0)	0.0 (0.0 – 100.0)	
	Management of dietary protein restriction	**Mean (SD)**	na	37.5 (17.7)	31.8 (25.3)	23.4 (22.4)	20.6 (17.4)	**0.048**
		**Median (Q1 – Q3)**	na	37.5 (25.0 – 50.0)	25.0 (0.0 – 100.0)	16.7 (0.0 – 95.8)	16.7 (0.0 – 70.8)	
	Practical impact of dietary protein restriction	**Mean (SD)**	na	34.5 (1.7)	33.2 (20.9)	32.4 (20.0)	30.7 (22.5)	0.860
		**Median (Q1 – Q3)**	na	34.5 (33.3 – 35.7)	32.1 (0.0 – 82.1)	30.4 (0.0 – 78.6)	28.6 (0.0 – 82.1)	
	Child food enjoyment	**Mean (SD)**	na	12.5 (17.7)	20.5 (20.8)	21.4 (28.3)	18.7 (21.5)	0.900
		**Median (Q1 – Q3)**	na	12.5 (0.0 – 25.0)	25.0 (0.0 – 75.0)	0.0 (0.0 – 100.0)	25.0 (0.0 – 75.0)	
	Guilt if dietary protein restriction not followed	**Mean (SD)**	na	50.0 (35.4)	47.6 (36.1)	46.7 (34.8)	41.5 (35.6)	0.716
		**Median (Q1 – Q3)**	na	50.0 (25.0 – 75.0)	50.0 (0.0 – 100.0)	50.0 (0.0 – 100.0)	25.0 (0.0 – 100.0)	

**Non-parametric P-value for between-group comparisons: Kruskal-Wallis*
In bold p-value<0.05
