# Supplementary material for: Assessment of the impact of phenylketonuria and its treatment on quality of life of patients and parents from seven European countries
Source: Orphanet J Rare Dis. 2015 Jun 18;10:80. doi: 10.1186/s13023-015-0294-x (PMC4542123; doi:10.1186/s13023-015-0294-x)
Supplement: Additional file 3: Table S3. — Comparisons of PKU-QOL scores according to BH4 intake. This file includes four tables presenting in the child, adolescent, adult and parent samples the comparison of PKU-QOL scores according to BH4 intake (BH4 intake; no BH4 intake). [file 13023_2015_294_MOESM3_ESM.pdf]

Additional File 3 : Comparison of PKU-QOL scores according to BH4 intake

Table 3_a : Comparison of the Child PKU-QOL scores according to BH4 intake

Modules	Domains		BH4 (N=27)	No BH4 (N=65)	p-value*
**Symptoms**	Headaches	**Mean (SD)**	15.7 (25.1)	15.2 (20.0)	0.778
		**Median (Q1 – Q3)**	0.0 (0.0 – 100.0)	0.0 (0.0 – 75.0)	
	Stomach aches	**Mean (SD)**	18.5 (23.6)	14.8 (22.1)	0.483
		**Median (Q1 – Q3)**	0.0 (0.0 – 75.0)	0.0 (0.0 – 100.0)	
	Tiredness	**Mean (SD)**	34.3 (29.5)	35.2 (25.2)	0.891
		**Median (Q1 – Q3)**	50.0 (0.0 – 100.0)	25.0 (0.0 – 100.0)	
	Irritability	**Mean (SD)**	22.2 (33.5)	17.2 (26.0)	0.800
		**Median (Q1 – Q3)**	0.0 (0.0 – 100.0)	0.0 (0.0 – 100.0)	
	Aggressiveness	**Mean (SD)**	13.0 (26.3)	11.9 (22.6)	0.936
		**Median (Q1 – Q3)**	0.0 (0.0 – 100.0)	0.0 (0.0 – 100.0)	
	Moodiness	**Mean (SD)**	23.1 (25.9)	13.5 (22.2)	0.091
		**Median (Q1 – Q3)**	0.0 (0.0 – 75.0)	0.0 (0.0 – 100.0)	
	Sadness	**Mean (SD)**	17.6 (22.8)	15.7 (23.6)	0.655
		**Median (Q1 – Q3)**	0.0 (0.0 – 75.0)	0.0 (0.0 – 100.0)	
	Anxiety	**Mean (SD)**	8.7 (18.6)	9.3 (18.2)	0.717
		**Median (Q1 – Q3)**	0.0 (0.0 – 50.0)	0.0 (0.0 – 75.0)	
	Lack of concentration	**Mean (SD)**	17.7 (27.1)	23.7 (28.8)	0.350
		**Median (Q1 – Q3)**	0.0 (0.0 – 100.0)	25.0 (0.0 – 100.0)	
	Slow thinking	**Mean (SD)**	24.0 (27.1)	22.3 (25.8)	0.802
		**Median (Q1 – Q3)**	25.0 (0.0 – 100.0)	25.0 (0.0 – 100.0)	
**PKU in general**	Emotional impact of PKU	**Mean (SD)**	32.1 (21.2)	33.3 (22.4)	0.850
		**Median (Q1 – Q3)**	33.3 (0.0 – 66.7)	33.3 (0.0 – 83.3)	
	Practical impact of PKU	**Mean (SD)**	12.0 (19.5)	10.5 (15.7)	0.908
		**Median (Q1 – Q3)**	0.0 (0.0 – 75.0)	0.0 (0.0 – 75.0)	
	Social impact of PKU	**Mean (SD)**	18.9 (21.9)	19.6 (16.4)	0.468
		**Median (Q1 – Q3)**	16.7 (0.0 – 91.7)	16.7 (0.0 – 75.0)	
	Overall impact of PKU	**Mean (SD)**	22.1 (16.7)	22.5 (14.8)	0.640
		**Median (Q1 – Q3)**	17.2 (0.0 – 71.9)	18.8 (0.0 – 78.1)	
	Anxiety - Blood test	**Mean (SD)**	13.4 (17.3)	11.9 (16.7)	0.531
		**Median (Q1 – Q3)**	12.5 (0.0 – 62.5)	0.0 (0.0 – 50.0)	
	Anxiety - Phe levels	**Mean (SD)**	53.7 (43.1)	41.1 (33.3)	0.219
		**Median (Q1 – Q3)**	50.0 (0.0 – 100.0)	25.0 (0.0 – 100.0)	
**Supplement administration**	Adherence to supplements	**Mean (SD)**	12.5 (15.9)	10.5 (17.7)	0.366
		**Median (Q1 – Q3)**	12.5 (0.0 – 50.0)	0.0 (0.0 – 100.0)	
	Practical impact of supplements	**Mean (SD)**	15.2 (29.9)	19.4 (32.1)	0.581
		**Median (Q1 – Q3)**	0.0 (0.0 – 100.0)	0.0 (0.0 – 100.0)	
	Guilt if poor adherence to supplements	**Mean (SD)**	31.0 (37.0)	50.4 (38.3)	**0.040**
		**Median (Q1 – Q3)**	25.0 (0.0 – 100.0)	50.0 (0.0 – 100.0)	
	Relationships within family because of supplements	**Mean (SD)**	12.0 (26.0)	8.5 (19.7)	0.808
		**Median (Q1 – Q3)**	0.0 (0.0 – 100.0)	0.0 (0.0 – 100.0)	
	Taste - Supplements	**Mean (SD)**	42.0 (27.2)	33.6 (33.5)	0.186
		**Median (Q1 – Q3)**	50.0 (0.0 – 100.0)	25.0 (0.0 – 100.0)	

Table 3_a : Comparison of the Child PKU-QOL scores according to BH4 intake (cont'd)

Modules	Domains		BH4 (N=27)	No BH4 (N=65)	p-value*
**Dietary protein restriction**	Food temptations	**Mean (SD)**	37.5 (30.4)	26.4 (26.4)	0.115
**Dietary protein restriction**		**Median (Q1 – Q3)**	50.0 (0.0 – 87.5)	25.0 (0.0 – 100.0)	
	Adherence to dietary protein restriction	**Mean (SD)**	9.9 (18.1)	11.1 (16.3)	0.510
		**Median (Q1 – Q3)**	0.0 (0.0 – 62.5)	0.0 (0.0 – 62.5)	
	Social impact of dietary protein restriction	**Mean (SD)**	21.5 (23.3)	17.4 (16.6)	0.819
		**Median (Q1 – Q3)**	12.5 (0.0 – 80.0)	15.0 (0.0 – 70.0)	
	Taste – low protein food	**Mean (SD)**	16.2 (19.6)	18.5 (24.4)	0.875
		**Median (Q1 – Q3)**	0.0 (0.0 – 50.0)	25.0 (0.0 – 100.0)	
	Food enjoyment	**Mean (SD)**	15.7 (21.0)	17.7 (24.5)	0.808
		**Median (Q1 – Q3)**	0.0 (0.0 – 50.0)	0.0 (0.0 – 100.0)	
	Guilt if dietary protein restriction not followed	**Mean (SD)**	44.6 (44.6)	55.0 (39.8)	0.281
		**Median (Q1 – Q3)**	50.0 (0.0 – 100.0)	50.0 (0.0 – 100.0)	
	Overall difficulty following dietary protein restriction	**Mean (SD)**	24.0 (36.5)	16.5 (23.5)	0.858
		**Median (Q1 – Q3)**	0.0 (0.0 – 100.0)	0.0 (0.0 – 100.0)	

**Non-parametric P-value for between-group comparisons: Mann-Whitney-Wilcoxon*
In bold p-value<0.05

Table 3_b : Comparison of the Adolescent PKU-QOL scores according to BH4 intake

Modules	Domains		BH4 (N=27)	No BH4 (N=83)	p-value*
**Symptoms**	Self-rated status health	**Mean (SD)**	33.3 (25.9)	35.3 (24.0)	0.680
		**Median (Q1 – Q3)**	25.0 (0.0 – 75.0)	50.0 (0.0 – 75.0)	
	Headaches	**Mean (SD)**	18.5 (23.6)	20.5 (26.0)	0.795
		**Median (Q1 – Q3)**	0.0 (0.0 – 75.0)	0.0 (0.0 – 100.0)	
	Stomach aches	**Mean (SD)**	14.8 (24.3)	14.4 (20.0)	0.802
		**Median (Q1 – Q3)**	0.0 (0.0 – 100.0)	0.0 (0.0 – 75.0)	
	Tiredness	**Mean (SD)**	40.7 (28.7)	40.4 (25.2)	0.911
		**Median (Q1 – Q3)**	50.0 (0.0 – 100.0)	50.0 (0.0 – 100.0)	
	Irritability	**Mean (SD)**	24.1 (26.4)	25.0 (25.8)	0.849
		**Median (Q1 – Q3)**	25.0 (0.0 – 75.0)	25.0 (0.0 – 100.0)	
	Aggressiveness	**Mean (SD)**	12.0 (22.3)	11.9 (22.8)	0.985
		**Median (Q1 – Q3)**	0.0 (0.0 – 75.0)	0.0 (0.0 – 100.0)	
	Moodiness	**Mean (SD)**	16.7 (19.6)	20.9 (23.7)	0.515
		**Median (Q1 – Q3)**	0.0 (0.0 – 50.0)	25.0 (0.0 – 75.0)	
	Sadness	**Mean (SD)**	4.6 (12.1)	14.7 (20.9)	**0.022**
		**Median (Q1 – Q3)**	0.0 (0.0 – 50.0)	0.0 (0.0 – 75.0)	
	Anxiety	**Mean (SD)**	13.0 (24.4)	14.4 (23.5)	0.694
		**Median (Q1 – Q3)**	0.0 (0.0 – 100.0)	0.0 (0.0 – 100.0)	
	Lack of concentration	**Mean (SD)**	17.6 (26.7)	20.3 (23.6)	0.424
		**Median (Q1 – Q3)**	0.0 (0.0 – 100.0)	12.5 (0.0 – 75.0)	
	Slow thinking	**Mean (SD)**	13.0 (18.8)	14.1 (20.2)	0.869
		**Median (Q1 – Q3)**	0.0 (0.0 – 50.0)	0.0 (0.0 – 75.0)	
**PKU in general**	Emotional impact of PKU	**Mean (SD)**	27.1 (15.3)	33.8 (20.9)	0.209
		**Median (Q1 – Q3)**	30.0 (0.0 – 70.0)	30.0 (0.0 – 90.0)	
	Practical impact of PKU	**Mean (SD)**	10.0 (12.9)	12.5 (14.2)	0.707
		**Median (Q1 – Q3)**	8.3 (0.0 – 33.3)	8.3 (0.0 – 50.0)	
	Social impact of PKU	**Mean (SD)**	11.2 (11.8)	17.1 (15.0)	0.052
		**Median (Q1 – Q3)**	8.3 (0.0 – 41.7)	16.7 (0.0 – 91.7)	
	Overall impact of PKU	**Mean (SD)**	17.8 (10.7)	24.4 (16.1)	0.066
		**Median (Q1 – Q3)**	17.5 (2.3 – 50.0)	22.7 (0.0 – 85.0)	
	Anxiety - Blood test	**Mean (SD)**	11.1 (21.9)	10.8 (20.4)	0.857
		**Median (Q1 – Q3)**	0.0 (0.0 - 87.5)	0.0 (0.0 – 100.0)	
	Anxiety - Phe levels	**Mean (SD)**	35.6 (33.3)	35.6 (31.3)	0.943
		**Median (Q1 – Q3)**	25.0 (0.0 – 100.0)	25.0 (0.0 – 100.0)	

Table 3_b : Comparison of the Adolescent PKU-QOL scores according to BH4 intake (cont'd)

Modules	Domains		BH4 (N=27)	No BH4 (N=83)	p-value*
**Supplement administration**	Adherence to supplements	**Mean (SD)**	7.6 (9.9)	16.7 (20.5)	0.103
		**Median (Q1 – Q3)**	3.1 (0.0 – 33.3)	7.3 (0.0 – 83.3)	
	Practical impact of supplements	**Mean (SD)**	6.6 (8.7)	22.6 (25.2)	**0.006**
		**Median (Q1 – Q3)**	3.1 (0.0 – 25.0)	12.5 (0.0 – 100.0)	
	Guilt if poor adherence to supplements	**Mean (SD)**	42.5 (33.5)	43.6 (36.6)	0.985
		**Median (Q1 – Q3)**	37.5 (0.0 – 100.0)	25.0 (0.0 – 100.0)	
	Relationships within family because of supplements	**Mean (SD)**	10.0 (17.0)	15.5 (27.0)	0.727
		**Median (Q1 – Q3)**	0.0 (0.0 – 50.0)	0.0 (0.0 – 100.0)	
	Taste - Supplements	**Mean (SD)**	41.3 (20.3)	40.9 (26.0)	0.644
		**Median (Q1 – Q3)**	50.0 (0.0 – 75.0)	50.0 (0.0 – 100.0)	
**Dietary protein restriction**	Food temptations	**Mean (SD)**	14.6 (18.8)	29.3 (29.3)	**0.049**
		**Median (Q1 – Q3)**	0.0 (0.0 – 50.0)	25.0 (0.0 – 100.0)	
	Adherence to dietary protein restriction	**Mean (SD)**	7.2 (10.6)	15.0 (19.8)	0.124
		**Median (Q1 – Q3)**	0.0 (0.0 – 33.3)	8.3 (0.0 – 83.3)	
	Practical impact of dietary protein restriction	**Mean (SD)**	22.8 (13.7)	28.1 (19.0)	0.574
		**Median (Q1 – Q3)**	20.7 (0.0 – 53.6)	28.6 (0.0 – 85.7)	
	Social impact of dietary protein restriction	**Mean (SD)**	5.3 (10.9)	16.0 (21.8)	**0.031**
		**Median (Q1 – Q3)**	0.0 (0.0 – 45.0)	7.5 (0.0 – 90.0)	
	Overall impact of dietary protein restriction	**Mean (SD)**	14.7 (11.2)	21.8 (18.0)	0.155
		**Median (Q1 – Q3)**	13.6 (0.0 – 37.5)	18.2 (0.0 – 86.4)	
	Taste – low protein food	**Mean (SD)**	22.9 (16.7)	26.4 (26.8)	0.978
		**Median (Q1 – Q3)**	25.0 (0.0 – 50.0)	25.0 (0.0 – 100.0)	
	Food enjoyment	**Mean (SD)**	11.8 (21.9)	17.1 (27.6)	0.482
		**Median (Q1 – Q3)**	0.0 (0.0 – 75.0)	0.0 (0.0 – 100.0)	
	Guilt if dietary protein restriction not followed	**Mean (SD)**	34.7 (36.5)	49.3 (34.9)	0.112
		**Median (Q1 – Q3)**	25.0 (0.0 – 100.0)	50.0 (0.0 – 100.0)	
	Overall difficulty following dietary protein restriction	**Mean (SD)**	3.9 (9.4)	20.3 (28.4)	**0.010**
		**Median (Q1 – Q3)**	0.0 (0.0 – 25.0)	0.0 (0.0 – 100.0)	

**Non-parametric P-value for between-group comparisons: Mann-Whitney-Wilcoxon*
In bold p-value<0.05

Table 3_c : Comparison of the Adult PKU-QOL scores according to BH4 intake

Modules	Domains		BH4 (N=15)	No BH4 (N=89)	p-value*
**Symptoms**	Self-rated health status	**Mean (SD)**	28.6 (25.7)	34.3 (22.4)	0.430
		**Median (Q1 – Q3)**	25.0 (0.0 – 75.0)	25.0 (0.0 – 100.0)	
	Headaches	**Mean (SD)**	17.9 (20.6)	21.9 (24.2)	0.654
		**Median (Q1 – Q3)**	12.5 (0.0 – 50.0)	25.0 (0.0 – 75.0)	
	Stomach aches	**Mean (SD)**	12.5 (23.5)	17.3 (26.1)	0.533
		**Median (Q1 – Q3)**	0.0 (0.0 – 75.0)	0.0 (0.0 – 100.0)	
	Tiredness	**Mean (SD)**	37.5 (27.3)	45.5 (27.5)	0.455
		**Median (Q1 – Q3)**	50.0 (0.0 – 75.0)	50.0 (0.0 – 100.0)	
	Trembling hands	**Mean (SD)**	10.7 (18.9)	12.8 (23.7)	0.975
		**Median (Q1 – Q3)**	0.0 (0.0 – 50.0)	0.0 (0.0 – 100.0)	
	Irritability	**Mean (SD)**	25.0 (21.9)	34.8 (25.2)	0.180
		**Median (Q1 – Q3)**	25.0 (0.0 – 50.0)	50.0 (0.0 – 100.0)	
	Aggressiveness	**Mean (SD)**	5.4 (10.6)	13.6 (22.4)	0.294
		**Median (Q1 – Q3)**	0.0 (0.0 – 25.0)	0.0 (0.0 – 75.0)	
	Moodiness	**Mean (SD)**	19.6 (22.3)	23.6 (25.2)	0.650
		**Median (Q1 – Q3)**	12.5 (0.0 – 50.0)	25.0 (0.0 – 75.0)	
	Sadness	**Mean (SD)**	26.8 (30.2)	29.3 (28.2)	0.687
		**Median (Q1 – Q3)**	25.0 (0.0 – 100.0)	25.0 (0.0 – 100.0)	
	Anxiety	**Mean (SD)**	23.2 (28.5)	21.9 (29.6)	0.751
		**Median (Q1 – Q3)**	12.5 (0.0 – 75.0)	0.0 (0.0 – 100.0)	
	Lack of concentration	**Mean (SD)**	23.2 (24.9)	24.7 (26.4)	0.914
		**Median (Q1 – Q3)**	25.0 (0.0 – 75.0)	25.0 (0.0 – 100.0)	
	Slow thinking	**Mean (SD)**	7.1 (15.3)	19.9 (27.6)	0.098
		**Median (Q1 – Q3)**	0.0 (0.0 – 50.0)	0.0 (0.0 – 100.0)	
**PKU in general**	Emotional impact of PKU	**Mean (SD)**	42.5 (22.3)	44.9 (22.2)	0.927
		**Median (Q1 – Q3)**	45.0 (0.0 – 70.0)	45.0 (0.0 – 100.0)	
	Practical impact of PKU	**Mean (SD)**	12.7 (17.0)	18.6 (16.9)	0.204
		**Median (Q1 – Q3)**	8.3 (0.0 – 58.3)	16.7 (0.0 – 75.0)	
	Social impact of PKU	**Mean (SD)**	14.1 (9.1)	18.4 (16.2)	0.601
		**Median (Q1 – Q3)**	16.7 (0.0 – 31.3)	16.7 (0.0 – 68.8)	
	Overall impact of PKU	**Mean (SD)**	22.4 (12.0)	30.9 (15.7)	0.373
		**Median (Q1 – Q3)**	24.0 (8.3 – 33.3)	27.1 (2.1 – 68.8)	
	Anxiety - Blood test	**Mean (SD)**	9.6 (11.6)	10.8 (23.4)	0.308
		**Median (Q1 – Q3)**	0.0 (0.0 – 25.0)	0.0 (0.0 – 100.0)	
	Anxiety - Phe levels	**Mean (SD)**	39.3 (21.3)	36.2 (28.2)	0.559
		**Median (Q1 – Q3)**	37.5 (0.0 – 75.0)	25.0 (0.0 – 100.0)	
	Anxiety - Phe levels during pregnancy	**Mean (SD)**	80.6 (24.3)	80.8 (24.8)	0.959
		**Median (Q1 – Q3)**	100.0 (50.0 – 100.0)	100.0 (0.0 – 100.0)	
	Financial impact of PKU	**Mean (SD)**	3.6 (9.1)	21.3 (28.3)	**0.013**
		**Median (Q1 – Q3)**	0.0 (0.0 – 25.0)	0.0 (0.0 – 100.0)	
	Information on PKU	**Mean (SD)**	30.4 (20.0)	34.8 (24.9)	0.570
		**Median (Q1 – Q3)**	25.0 (0.0 – 75.0)	25.0 (0.0 – 100.0)	

Table 3_c : Comparison of the Adult PKU-QOL scores according to BH4 intake (cont'd)

Modules	Domains		BH4 (N=15)	No BH4 (N=89)	p-value*
**Supplement administration**	Adherence to supplements	**Mean (SD)**	11.1 (9.6)	20.8 (20.4)	0.567
		**Median (Q1 – Q3)**	16.7 (0.0 – 16.7)	16.7 (0.0 – 66.7)	
	Practical impact of supplements	**Mean (SD)**	9.4 (10.8)	22.6 (21.7)	0.257
		**Median (Q1 – Q3)**	6.3 (0.0 – 25.0)	18.8 (0.0 – 100.0)	
	Guilt if poor adherence to supplements	**Mean (SD)**	50.0 (28.9)	47.8 (33.3)	0.886
		**Median (Q1 – Q3)**	50.0 (25.0 – 75.0)	50.0 (0.0 – 100.0)	
	Relationships within family because of supplements	**Mean (SD)**	18.8 (37.5)	10.8 (21.4)	0.891
		**Median (Q1 – Q3)**	0.0 (0.0 – 75.0)	0.0 (0.0 – 100.0)	
	Taste - Supplements	**Mean (SD)**	50.0 (20.4)	49.7 (25.8)	0.926
		**Median (Q1 – Q3)**	50.0 (25.0 – 75.0)	50.0 (0.0 – 100.0)	
**Dietary protein restriction**	Food temptations	**Mean (SD)**	12.5 (12.5)	36.7 (24.8)	**0.027**
		**Median (Q1 – Q3)**	12.5 (0.0 – 25.0)	37.5 (0.0 – 100.0)	
	Adherence to dietary protein restriction	**Mean (SD)**	4.7 (9.4)	20.6 (18.2)	0.064
		**Median (Q1 – Q3)**	0.0 (0.0 – 18.8)	20.0 (0.0 – 56.3)	
	Social impact of dietary protein-restriction	**Mean (SD)**	2.7 (3.8)	19.0 (18.6)	**0.016**
		**Median (Q1 – Q3)**	0.0 (0.0 – 8.3)	15.8 (0.0 – 79.2)	
	Practical impact of dietary protein restriction	**Mean (SD)**	15.5 (14.5)	36.5 (19.6)	**0.019**
		**Median (Q1 – Q3)**	12.5 (0.0 – 39.3)	35.7 (0.0 – 75.0)	
	Overall impact of dietary protein restriction	**Mean (SD)**	9.5 (9.4)	27.7 (17.1)	**0.016**
		**Median (Q1 – Q3)**	6.8 (0.0 – 25.0)	26.9 (0.0 – 75.0)	
	Taste – low protein food	**Mean (SD)**	50.0 (.)	33.1 (21.4)	0.292
		**Median (Q1 – Q3)**	50.0 (50.0 – 50.0)	25.0 (0.0 – 100.0)	
	Food enjoyment	**Mean (SD)**	10.0 (13.7)	27.2 (30.0)	0.223
		**Median (Q1 – Q3)**	0.0 (0.0 – 25.0)	25.0 (0.0 – 100.0)	
	Guilt if dietary protein restriction not followed	**Mean (SD)**	65.0 (28.5)	53.2 (31.9)	0.415
		**Median (Q1 – Q3)**	75.0 (25.0 – 100.0)	50.0 (0.0 – 100.0)	
	Overall difficulty following dietary protein restriction	**Mean (SD)**	16.7 (30.3)	29.1 (27.9)	0.232
		**Median (Q1 – Q3)**	0.0 (0.0 – 75.0)	25.0 (0.0 – 100.0)	

**Non-parametric P-value for between-group comparisons: Mann-Whitney-Wilcoxon*
In bold p-value<0.05

Table 3_d : Comparison of the Parent PKU-QOL scores according to patient's BH4 intake

Modules	Domains		BH4 (N=67)	No BH4 (N=186)	p-value*
**Symptoms**	Child health status	**Mean (SD)**	28.0 (22.4)	32.5 (24.4)	0.221
		**Median (Q1 – Q3)**	25.0 (0.0 – 75.0)	25.0 (0.0 – 100.0)	
	Headaches	**Mean (SD)**	13.8 (21.0)	14.8 (20.7)	0.624
		**Median (Q1 – Q3)**	0.0 (0.0 – 75.0)	0.0 (0.0 – 75.0)	
	Stomach aches	**Mean (SD)**	23.5 (26.8)	17.3 (23.2)	0.107
		**Median (Q1 – Q3)**	25.0 (0.0 – 75.0)	0.0 (0.0 – 100.0)	
	Tiredness	**Mean (SD)**	32.1 (25.3)	31.9 (24.9)	0.926
		**Median (Q1 – Q3)**	25.0 (0.0 – 100.0)	25.0 (0.0 – 100.0)	
	Irritability	**Mean (SD)**	38.1 (28.7)	35.2 (27.1)	0.523
		**Median (Q1 – Q3)**	50.0 (0.0 – 100.0)	25.0 (0.0 – 100.0)	
	Aggressiveness	**Mean (SD)**	14.4 (22.0)	12.4 (21.8)	0.443
		**Median (Q1 – Q3)**	0.0 (0.0 – 75.0)	0.0 (0.0 – 100.0)	
	Moodiness	**Mean (SD)**	34.1 (27.6)	25.3 (24.6)	**0.023**
		**Median (Q1 – Q3)**	25.0 (0.0 – 100.0)	25.0 (0.0 – 100.0)	
	Sadness	**Mean (SD)**	18.2 (22.6)	18.2 (20.8)	0.819
		**Median (Q1 – Q3)**	0.0 (0.0 – 100.0)	25.0 (0.0 – 100.0)	
	Anxiety	**Mean (SD)**	14.8 (21.7)	14.0 (21.9)	0.708
		**Median (Q1 – Q3)**	0.0 (0.0 – 75.0)	0.0 (0.0 – 100.0)	
	Lack of concentration	**Mean (SD)**	34.1 (28.6)	29.0 (28.1)	0.192
		**Median (Q1 – Q3)**	25.0 (0.0 – 100.0)	25.0 (0.0 – 100.0)	
	Slow thinking	**Mean (SD)**	20.8 (25.8)	19.6 (26.8)	0.611
		**Median (Q1 – Q3)**	0.0 (0.0 – 75.0)	0.0 (0.0 – 100.0)	
**PKU in general**	Emotional impact of PKU	**Mean (SD)**	40.9 (25.3)	43.1 (22.1)	0.381
		**Median (Q1 – Q3)**	43.8 (0.0 – 93.8)	37.5 (0.0 – 100.0)	
	Practical impact of PKU	**Mean (SD)**	11.4 (12.9)	15.3 (14.8)	0.061
		**Median (Q1 – Q3)**	4.2 (0.0 – 41.7)	12.5 (0.0 – 70.0)	
	Social impact of PKU	**Mean (SD)**	15.8 (15.9)	17.5 (17.0)	0.346
		**Median (Q1 – Q3)**	10.0 (0.0 – 55.0)	12.5 (0.0 – 90.0)	
	Overall impact of PKU	**Mean (SD)**	21.4 (15.3)	24.7 (15.5)	0.083
		**Median (Q1 – Q3)**	18.8 (0.0 – 56.7)	21.7 (0.0 – 76.9)	
	Child anxiety - Blood test	**Mean (SD)**	23.3 (27.4)	23.7 (28.0)	0.981
		**Median (Q1 – Q3)**	12.5 (0.0 – 100.0)	12.5 (0.0 – 100.0)	
	Impact of child anxiety - Blood test	**Mean (SD)**	21.9 (27.1)	25.6 (29.1)	0.386
		**Median (Q1 – Q3)**	12.5 (0.0 – 100.0)	12.5 (0.0 – 100.0)	
	Anxiety - Phe levels	**Mean (SD)**	60.2 (33.7)	52.9 (31.0)	0.113
		**Median (Q1 – Q3)**	75.0 (0.0 – 100.0)	50.0 (0.0 – 100.0)	
	Financial impact of PKU	**Mean (SD)**	24.2 (28.6)	27.2 (29.1)	0.428
		**Median (Q1 – Q3)**	25.0 (0.0 – 100.0)	25.0 (0.0 – 100.0)	
	Information on PKU	**Mean (SD)**	27.3 (20.0)	32.6 (25.7)	0.178
		**Median (Q1 – Q3)**	25.0 (0.0 – 100.0)	25.0 (0.0 – 100.0)	

Table 3_d : Comparison of the Parent PKU-QOL scores according to patient's BH4 intake (cont'd)

Modules	Domains		BH4 Mean (SD) (N=67)	No BH4 Mean (SD) (N=186)	p-value*
**Supplement administration**	Adherence to supplements	**Mean (SD)**	9.6 (13.3)	12.4 (19.3)	0.681
		**Median (Q1 – Q3)**	0.0 (0.0 – 50.0)	0.0 (0.0 – 100.0)	
	Management of supplements	**Mean (SD)**	12.8 (26.0)	18.8 (27.7)	0.077
		**Median (Q1 – Q3)**	0.0 (0.0 – 100.0)	0.0 (0.0 – 100.0)	
	Practical impact of supplements	**Mean (SD)**	16.8 (24.3)	21.6 (23.2)	0.113
		**Median (Q1 – Q3)**	8.3 (0.0 – 100.0)	16.7 (0.0 – 100.0)	
	Guilt if poor adherence to supplements	**Mean (SD)**	51.6 (33.9)	52.7 (34.2)	0.849
		**Median (Q1 – Q3)**	50.0 (0.0 – 100.0)	50.0 (0.0 – 100.0)	
	Relationships within family because of supplements	**Mean (SD)**	15.1 (24.0)	20.6 (26.8)	0.189
		**Median (Q1 – Q3)**	0.0 (0.0 – 75.0)	0.0 (0.0 – 100.0)	
**Dietary protein restriction**	Adherence to dietary protein restriction	**Mean (SD)**	8.7 (20.1)	15.1 (26.2)	0.102
		**Median (Q1 – Q3)**	0.0 (0.0 – 100.0)	0.0 (0.0 – 100.0)	
	Management of dietary protein restriction	**Mean (SD)**	24.0 (21.6)	24.8 (22.2)	0.906
		**Median (Q1 – Q3)**	20.8 (0.0 – 83.3)	20.8 (0.0 – 100.0)	
	Practical impact of dietary protein restriction	**Mean (SD)**	29.7 (18.1)	32.5 (21.5)	0.537
		**Median (Q1 – Q3)**	28.6 (0.0 – 64.3)	28.6 (0.0 – 82.1)	
	Child food enjoyment	**Mean (SD)**	19.7 (21.5)	20.5 (25.0)	0.812
		**Median (Q1 – Q3)**	25.0 (0.0 – 100.0)	25.0 (0.0 – 100.0)	
	Guilt if dietary protein restriction not followed	**Mean (SD)**	38.0 (34.7)	47.3 (35.1)	0.091
		**Median (Q1 – Q3)**	25.0 (0.0 – 100.0)	50.0 (0.0 – 100.0)	

**Non-parametric P-value for between-group comparisons: Mann-Whitney-Wilcoxon*
In bold p-value<0.05
